# Integrated reiterative pipeline for rapid epitope-based pan-alphavirus vaccines

**DOI:** 10.1126/sciadv.aeb2066

**Published:** 2026-03-11

**Authors:** Alice F. Versiani, Peter McCaffrey, Helder V. Ribeiro-Filho, Natalia I. O. Silva, Paulo S. Lopes-de-Oliveira, Jean-Paul Carrera, Mauricio L. Nogueira, Rafael E. Marques, Shannan L. Rossi, Nikos Vasilakis

**Affiliations:** ^1^ Department of Pathology, University of Texas Medical Branch, 301 University Blvd. Galveston, TX 77555-0609, USA.; ^2^ Brazilian Biosciences National Laboratory - LNBio, Brazilian Center for Research in Energy and Materials - CNPEM, Campinas 13083-100, SP, Brazil.; ^3^ Department of Research in Virology and Biotechnology, Gorgas Memorial Institute of Health Studies, Panama City, Panama.; ^4^ Departamento de Doenças Infecciosas, Faculdade de Medicina de São José do Rio Preto, São José do Rio Preto, SP, Brazil.; ^5^ Institute for Human Infection and Immunity, University of Texas Medical Branch, 301 University Blvd. Galveston, TX 77555-0610, USA.; ^6^ Center for Vector-Borne and Zoonotic Diseases, University of Texas Medical Branch, 301 University Blvd. Galveston, TX 77555-0609, USA.

## Abstract

The vast diversity of the virosphere underscores the need for rapid, adaptable vaccine development infrastructures. Arthropod-borne zoonotic alphaviruses, in particular, continue to pose substantial threats to human and animal health. We present a fast, multitarget vaccine design pipeline integrating machine learning–based epitope prediction, protein modeling, and docking to prioritize viral peptides by immunogenicity, allele coverage, solubility, and stability. T cell epitopes were validated using peptide microarrays and molecular dynamics simulations, confirming receptor binding accuracy. Flow cytometry of murine and human peripheral blood mononuclear cells demonstrated robust T cell activation and cytokine secretion (IFN-γ, TNF-α, or IL-2), dependent on species and HLA allele. Final candidates were selected by composite immunogenicity scores. While this study primarily validates the T cell–specific arm of our predictive pipeline, complementary B cell epitope analyses are ongoing. Our findings support the development of broadly protective pan-alphaviral vaccines and the establishment of efficient, tunable processes for global vaccine development.

## Teaser

A computational, in vitro, and ex vivo pipeline enabling sustainable efficiency from viral proteome to vaccine payload.

## INTRODUCTION

Emerging viral diseases have devastated human populations for millennia. The 1918 influenza pandemic killed an estimated 1% of the global population (*1*), and the long-term impact of severe acute respiratory syndrome coronavirus 2 (SARS-CoV-2) on global health and economies is yet to be elucidated (*2*, *3*). Vaccination plays a crucial role in mitigating the disastrous effects of infectious diseases. Vaccines for SARS-CoV-2 were developed from genetic profile to functional candidates with unprecedented speed. However, this success stemmed from an intensive and potentially irreplicable global concentration of resources and effort. Preventive measures for emerging and reemerging pathogens must be in place to preempt future pandemics.

Recent advances in computational biology and machine learning are improving the development of rationally designed vaccines. Epitope-based designs simplify synthesis while enhancing stability, solubility, safety, and plasticity for delivery methods and formulations (*4*). In silico tools are leveraged to select short immunogenic peptide fragments to elicit strong and targeted immune responses and amplify antigen coverage. Similar approaches have been used to identify prototype candidates for *Leishmania* sp. (*5*, *6*), hepatitis C virus (*7*), SARS-CoV-2 (*8*), Mayaro virus (MAYV) (*9*), Marburg virus (*10*), and influenza virus (*11*). This approach enables multitarget designs to enhance immunogenicity and reduce toxicity and allergenicity. In addition, immunoinformatic tools can be used to stimulate specific B and T cell responses. The efficiency of the candidate vaccine to trigger an effective immune response can also be assessed by an in silico immune stimulation using structural modeling, refinement, and validation tools (*12*, *13*). Examples of successful computationally designed immunogen candidates now in clinical evaluation include the personalized neoantigen vaccines V940 (Moderna/Merck) and GRANITE (Gritstone Bio), which are in phase 3 and phase 2 trials, respectively (*14*, *15*). As potential emerging pathogens, alphaviruses are positive-sense, single-stranded RNA viruses transmitted by mosquito vectors. Medically important alphaviruses can be segregated into encephalitic or arthritogenic. Arthritogenic alphaviruses like chikungunya virus (CHIKV) and MAYV cause inflammatory musculoskeletal and joint-associated diseases, often with chronic pain affecting quality of life. Encephalitic alphaviruses include Eastern (EEEV), Western (WEEV), and Venezuelan (VEEV) equine encephalitis viruses can lead to neurological manifestations (*16*, *17*). The expansive geographic distribution, diversity of vectors, and breadth of reservoirs for these viruses, as well as anthropogenic and ecological factors, provide ample opportunity for their emergence into humans and animals [reviewed in (*18*, *19*)]. Although investments have been made to develop vaccines against these pathogens [reviewed in (*20*)], US Food and Drug Administration (FDA) approval for the first alphavirus (CHIKV) vaccine was not issued until 2023 (*21*). The cocirculation of alphaviruses, recent outbreaks, high morbidity and mortality of viral family members, and lack of therapeutics (*22*) underscore the need for multivalent immunogens.

Twin efforts are required to (i) bolster genomic surveillance of emerging pathogens and (ii) develop a frictionless process to generate vaccine candidates tuned to these data. Here, we focus on creating a system for iterative vaccine design that can be scalable, easily revised, and broadly accessible for various surveillance initiatives. Specifically, we describe a previously unknown pipeline to engineer immunogens that target genetically diverse alphaviruses. We demonstrate the computational, in vitro, and in vivo processes for an efficient, sustainable development pipeline from viral proteome to candidate vaccine payload.

## RESULTS

### Comprehensive in silico analysis of MHC-I and MHC-II peptide signatures indicates overlap among alphaviruses

**Fig. 1.**
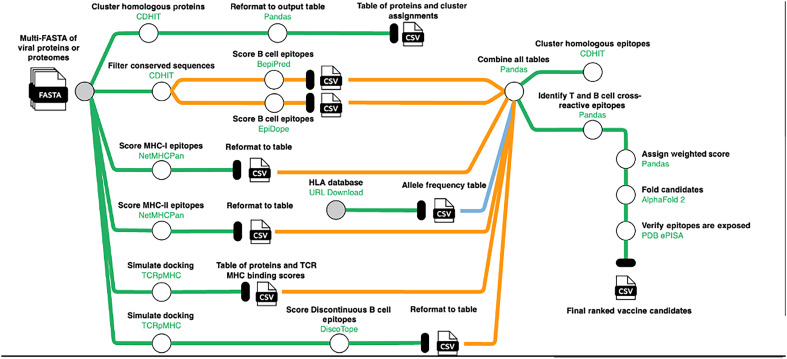
Epitope selection model. The schematic shows all steps required for the B cell epitope profiling, T cell epitope profiling, and vaccine candidate design. Green lines represent the flow of data between pipeline steps, and orange lines indicate that data formats have been converted or that assets have been split (as with conserved sequences being duplicated for evaluation by BepiPred and EpiDope). Blue lines indicate that assets have been newly introduced into the pipeline (because the HLA frequency table is generated and joined to the pipeline midstream). White circles represent processing steps, and gray circles represent external data sources that must be provided to the pipeline (these are the multi-FASTA files representing the viral proteomes and the HLA frequency database). Each step is labeled with a description (in black text) and the name of the primary tool used (in green text).

The vaccine design pipeline begins with a collection of target proteomes representing the viruses against which the vaccine is intended to be protective (table S1). The pipeline consists of three core design phases: B cell epitope profiling, T cell epitope profiling, and vaccine candidate design (Fig. 1). For B cell profiling, the pipeline accepts a list of UniProt accessions or protein sequences that represent targets against which effective antibodies would be raised (i.e., effective targets of neutralizing antibodies). From here, the initial proteomes are BLASTed against each submitted target protein, and multiple sequence alignment is performed to identify the conserved protein sequences from among the proteomes and the target sequences. These are then submitted to epitope detection using EpiDope and BepiPred. For T cell profiling, the submitted proteomes undergo epitope prediction for both major histocompatibility complex I (MHC-I) and MHC-II epitopes using netMHCpan and all alleles common to the target vaccine region. Following this, epitopes whose predicted binding affinity is in the highest 5% of all scored peptides are carried forward in a model of the TCRpMHC complex for each epitope. Last, free solvation energy and surface area (both derived from TCRpMHC models) as well as predicted binding affinity and allele frequency are combined to create a weighted immunogenicity score. The weighted score is calculated as the sum of each allele frequency multiplied by the allele-specific binding affinity, thus producing a weighted binding affinity. Last, for vaccine candidate design, scored B and T cell epitopes are submitted to JessEV to render vaccine candidate designs. For example, starting from an initial collection of 114 alphaviral proteins, the pipeline extracts 90,472 candidate MHC-I epitopes, 110,158 candidate MHC-II epitopes, and 3256 candidate B cell epitopes. This ultimately results in 98 epitopes that go into final vaccine design with JessEV (*23*).

### Peptide microarray indicates overlapping MHC-I and MHC-II epitopes among encephalitic and arthritogenic alphaviruses

Intending to improve the pipeline selection of the most immunogenic pan-alphavirus peptides among 726 epitope candidates, we assessed peptide reactivity in vitro using a slide microarray (PEPperPRINT, Heidelberg, Germany) tested against preexposed sera from infected human, mouse, or nonhuman primates (NHPs). Human samples were selected from cohorts infected with Madariaga virus (MADV), VEEV, and CHIKV. Mouse sera biobanks included not only CHIKV, MAYV, and VEEV-infected samples but also sera from animals vaccinated with TC-83, a VEEV live-attenuated vaccine commonly used as a surrogate in VEEV pathogenesis studies (*24*). Last, the NHP sera biobank includes CHIKV-infected samples and Zika virus serum samples from an off-target control to another arbovirus (table S2).

**Fig. 2.**
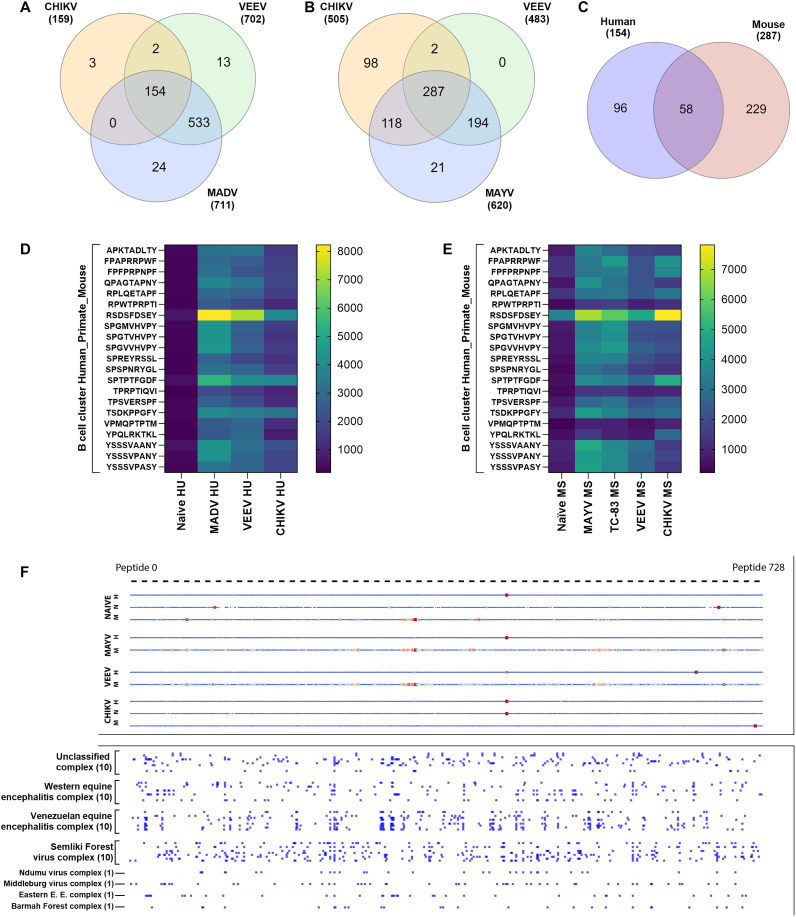
Panoramic evaluation of the reactivity of pan-alphavirus epitopes tested against preexposed sera. Peptide microarray was performed with samples originating from humans, mice, and NHPs. Data are presented by median fluorescence after background subtraction. Overlapping pan-alphaviruses epitopes were identified by the relative changes of the fluorescence intensity (*z*-score) on (**A**) human and (**B**) murine Venn diagrams. (**C**) Overlapping pan-alphaviruses epitopes among human and murine responses. (**D** and **E**) Heatmaps of B-cluster epitopes fluorescent signal against human and mouse sera, respectively. (**F**) A representative panel of all 726 tested peptides. Warmer colors represent higher fluorescence signals. The bottom panel identifies in silico predictions of overlapping epitopes among all alphavirus complexes. Unclassified complex: Salmon Pancreas Disease virus, Sleeping Disease virus, Tai Forest virus, MADV, Caaingua virus, Tonate virus, Southern Elephant Seal virus, Salmonid alphavirus, Mwinilunga virus, Eilat virus. Western equine encephalitis complex: WEEV, Highlands J virus, Fort Morgan virus, Buggy Creek virus, Whataroa virus, Ockelpo virus, Sindbis virus, Kyzylagach virus, Babanki virus, and Aura virus. Venezuelan equine encephalitis complex: VEEV, Bijou Bridge virus, Trocara virus, Rio Negro virus, Pixuna virus, Maramana virus, Mucambo virus, Masso das Pedras virus, Everglades virus, and Cabassou virus. Semliki Forest virus complex: Me Tri Virus, Semliki Forest virus, Sagiyama virus, Ross River virus, Igbo-Ora virus, Onyong-nyong virus, Una virus, Mayaro virus, Getah virus, chikungunya virus, and Bebaru virus.

As expected, most of the epitopes are recognized by humoral responses elicited by two or more viruses for either species tested (Fig. 2, A, B, and F). In an overall analysis of the most reactive epitopes, we can observe clustered areas for both human, mouse, and NHP-positive samples (fig. S1). None of the tested naïve sera presented significant hits. Expectedly, both encephalitic viruses evaluated with human samples (MADV and VEEV) have the highest number of common reactive epitopes (*n* = 533) (Fig. 2A). However, the pipeline was able to select more than 100 highly reactive epitopes (*z*-score > 1.0) for both tested species and viruses. Among overlapping epitopes, we found 154 pan-alphavirus epitopes for humans and 257 for mice. From those, 58 epitopes were considered reactive against all viruses tested on both species (Fig. 2C). The pipeline demonstrated accurate epitope selection as the lowest-ranked epitopes showed no reactivity with positive sera from any species or viruses, except for one epitope in the human sera pools (fig. S2). Although not the main focus of the pipeline, epitope sequences that cluster on both T and B cell receptors were identified, with epitopes not only crossing the pan-alpha spectra but also being reactive for more than one species, characterizing a good candidate for the final peptide selection (Fig. 2, D and E). Our vaccine design pipeline primarily prioritizes T cell epitopes while incorporating predicted B cell epitopes to enhance vaccine versatility and breadth of immune protection. Rather than performing extensive B cell epitope optimization, we score and select B cell epitopes on the basis of protein targets relevant to antibody responses and track both linear and discontinuous epitopes predicted to cross-react with selected T cell targets. Last, top-ranked epitopes with high binding affinity to MHC-I (*n* = 9) and MHC-II (*n* = 8) receptors were selected and synthesized for in vitro validation. Most of these peptides are included in the Envelope protein complex sequence of alphaviruses. A full description of receptor type, classification, species selection, and virus root can be found in table S3.

### MD simulations agree with in silico and in vitro predictions from the pipeline

To assess the feasibility of the identified peptides to bind and be presented in MHC-I or MHC-II from a structural biology perspective, we used molecular modeling and molecular dynamics (MD) simulations. Initially, we modeled the peptides into two prevalent human lymphocyte antigens (HLAs): HLA A*02:01:01:01 for MHC-I and HLA-DRA1:DRB1*07:01:01:01 for MHC-II, using two AlphaFold-based approaches: a customized version of AlphaFold2-multimer (AF2M) version (*25*) and ColabFold (*26*). Subsequently, we performed MD simulations to evaluate the stability of the peptide in the MHC binding site. For the MHC-I peptides, seven of the nine identified peptides (MHCI-pep1, MHCI-pep2, MHCI-pep3, MHCI-pep4, MHCI-pep5, MHCI-pep6, and MHCI-pep9) were successfully modeled by AF2M or ColabFold, exhibiting a canonical binding mode, as assessed by estimating the deviation from known peptide-bound three-dimensional (3D) structures (table S4). These seven peptides displayed appropriate anchors at the P2 and PΩ MHC-I binding sites. The remaining two peptides (MHCI-pep7 and MHCI-pep8) did not exhibit correct placement of the peptide C terminus to the MHC-I PΩ binding site in our models. In addition to the proper binding mode, MHCI-pep1, MHCI-pep4, and MHCI-pep5 achieved high-confidence modeling scores (>0.85) (table S4).

**Fig. 3.**
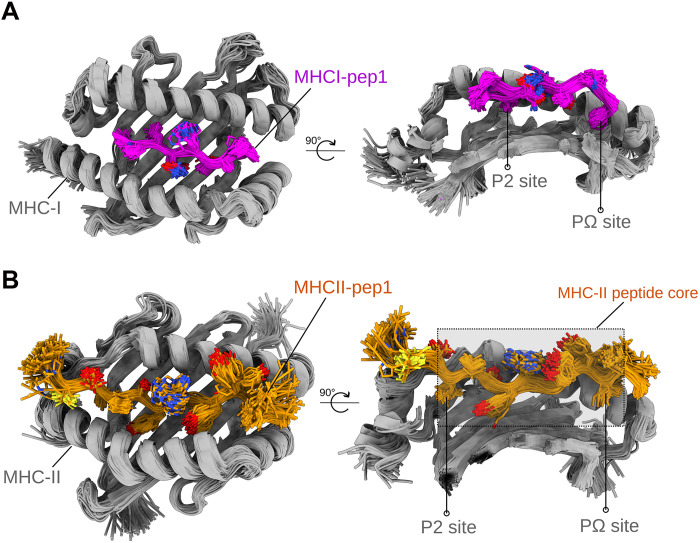
Representative superposed trajectory of MD simulations from two different identified peptides bound to MHC-I or MHC-II molecules. (**A**) Superposed trajectory of MHCI-pep1 bound to the MHC-I (HLA A*02:01:01:01). The presented trajectory comprises 100 superposed frames from a single replica of 200 ns after superposition by the MHC molecule. The peptide is depicted in purple cartoon and sticks, while the MHC molecule is depicted as a gray cartoon. The left panel shows an upper view of the complex, and the right panel shows a side view (for clarity, part of the MHC molecule is not shown). The MHC P2 and PΩ anchoring sites are indicated. (**B**) Same representation as in (A) but showing the trajectory of MHCII-pep1 bound to the MHC-II (HLA-DRA1: DRB1*07:01:01:01). The MHC-II peptide core is highlighted to indicate the peptide regions that fit into the MHC groove. Images of the structures were created using ChimeraX program (*80*).

Through 200-ns MD simulations across five independent replicas, initiated from the modeled complexes, we observed that the seven peptides with a canonical binding mode were generally stable, maintaining the MHC-I–anchored sites in most replicas. Notably, MHCI-pep1, MHCI-pep5, and MHCI-pep6 exhibited the lowest deviation along the trajectory [median peptide backbone root mean square deviation (RMSD) of 2.11, 1.48, and 1.17, respectively, considering the last half of the trajectory] in all evaluated replicas (fig. S3), consistent with the deviation observed in known binder peptides (fig. S4). As a representative case, Fig. 3 illustrates the ensemble of MHCI-pep1 conformations obtained from the simulated trajectory.

For the MHC-II peptides, six of the eight peptides were successfully modeled by AF2M or ColabFold with a canonical binding mode (table S4). Two peptides (MHCII-pep4 and MHCII-pep8) did not exhibit appropriate fitting of the C terminus into the MHC-II groove in our models. Because modeling peptides bound to MHC-II is typically more challenging than MHC-I cases due to their increased susceptibility to positional shifting, we used an orthogonal method and compared the peptide binding core predicted by NetMHCIIpan (*27*) with the core observed in the 3D models. NetMHCIIpan predictions corroborated the binding core of four peptides: MHCII-pep1, MHCII-pep5, MHCII-pep6, and MHCII-pep7, thereby supporting confidence in the models (table S5). All six modeled peptides that exhibited canonical binding modes also demonstrated high backbone stability at the peptide core in the MD simulations (fig. S5). As a representative case, Fig. 3 illustrates the ensemble of the MHCII-pep1 conformations obtained from the simulated trajectory. Together, these structure-based in silico results suggest the feasibility of most peptides identified in the pipeline to correctly bind to MHC-I and MHC-II alleles, assuming canonical binding modes. Furthermore, the MD simulations indicated that most peptides stably bind to the MHC grooves, maintaining the anchored peptide positions at MHC binding sites.

### The immunogenicity profile of preexposed murine PBMCs restimulated with candidate peptides indicates a strong T cell activation and IFN-γ secretion

**Fig. 4.**
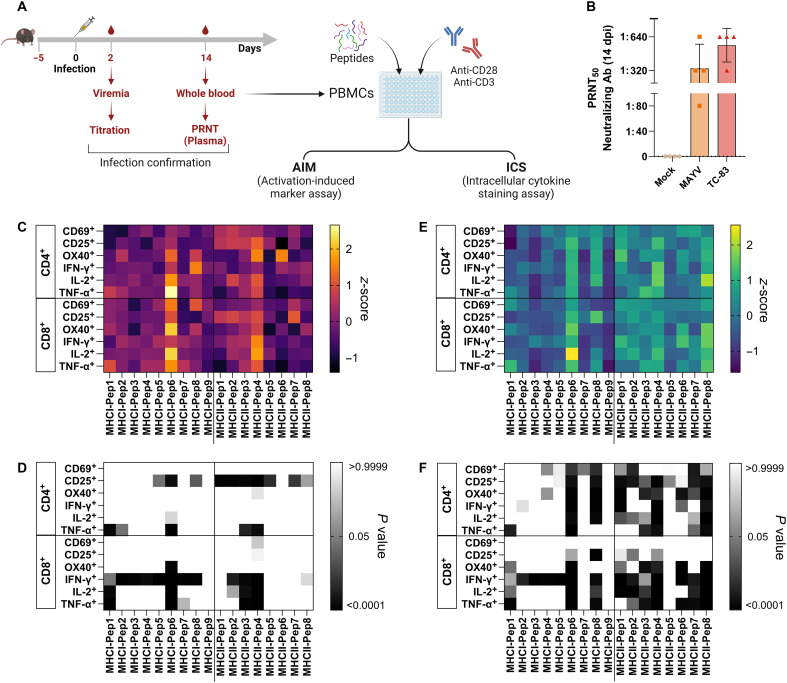
Peptide induced T cell reactivity on murine PBMCs preexposed to alphaviruses. (**A**) Schematic design of mouse infection with MAYV and TC-83. Blood was collected on days 2 and 14 postinfection for confirmation. Isolated PBMCs were restimulated in vitro and evaluated by flow cytometry for T cell activation (CD4^+^/CD8^+^) by surface markers (CD69, CD5, and OX-40) and secretion of cytokines (IFN-γ, TNF-α, and IL-2). (**B**) PRNT_50_ confirms the secretion of neutralizing antibodies after infection with both MAYV and TC-83. (**C**) Relative changes (*z*-score) of surface markers and cytokines on MAYV preexposed PBMCs after restimulation with peptides. (**D**) Heatmap with the representation of *P* values obtained by two-way analysis of variance (ANOVA) statistical analysis of (C). (**E**) Relative changes (*z*-score) of surface markers and cytokines on TC-83 preexposed PBMCs after restimulation with peptides. (**F**) Heatmap with the representation of *P* values obtained by two-way ANOVA with Dunnett’s multiple comparisons test of (E).

To validate the selected peptides for a T cell antigen–specific immunogenic response, we infected mice with MAYV, a representative of the arthritogenic alphaviruses, or with TC-83, a representative of the encephalitic alphaviruses. Later, the isolated peripheral blood mononuclear cell (PBMC) was restimulated ex vivo. We detected activated and effector T cell cytokine expression using flow cytometry (Fig. 4A). Successful infection and promotion of an immune response against the viruses were confirmed using the plaque reduction neutralization test assay (PRNT_50_, Fig. 4B). We selected surface activation-induced markers that indicate early (CD69), moderate late (CD25), and late T cell activation (OX-40). To further characterize the functionality of the activated T cells, we measured the induction of cytokines that are central for T cell priming, proliferation, and differentiation, such as interferon-γ (IFN-γ), tumor necrosis factor–α (TNF-α), and interleukin-2 (IL-2).

We observed that both sets of peptides, with binding affinity to MHC-I or MHC-II, were able to stimulate T cell activation. However, in PBMC originating from MAYV-infected mice (Fig. 4, C and D), the response was dominated by CD8^+^ cells secreting IFN-γ and activated CD4^+^ cells (CD25^+^). Specifically, peptides MHC-I pep1, MHC-I pep 6, MHC-II pep 3, and MHC-II pep 4 promoted synergy of responses on activated T CD8^+^ cells secreting IFN-γ and IL-2 and activated T CD4^+^ cells secreting TNF-α. In the case of TC-83 immunogenicity, all MHC-II peptides (apart from MHC-II pep5) stimulated a statistically significant response compared with the untreated (UT) control, especially considering increases in lymphocytes expressing mid and late activation markers (CD25 and OX-40, respectively). The response against MHC-I peptides’ stimulation in PBMC originating from TC-83 infected mice was similar to that of MAYV-infected mice, dominated by a strong IFN-γ expression on T CD8^+^ cells. Complete data plotted by positive-cell percentage can be observed in fig. S6, including experimental controls. The peptides that stimulated the most significant responses in murine PBMC (MHC-I pep1, MHC-I pep 6, MHC-II pep 3, and MHC-II pep 4) were identified in the pipeline as top-ranked for both human and mouse MHC alleles (table S3).

### Pan-alphavirus peptides are immunogenic in human PBMCs, but T cell activation varies depending on HLA allele groups

**Fig. 5.**
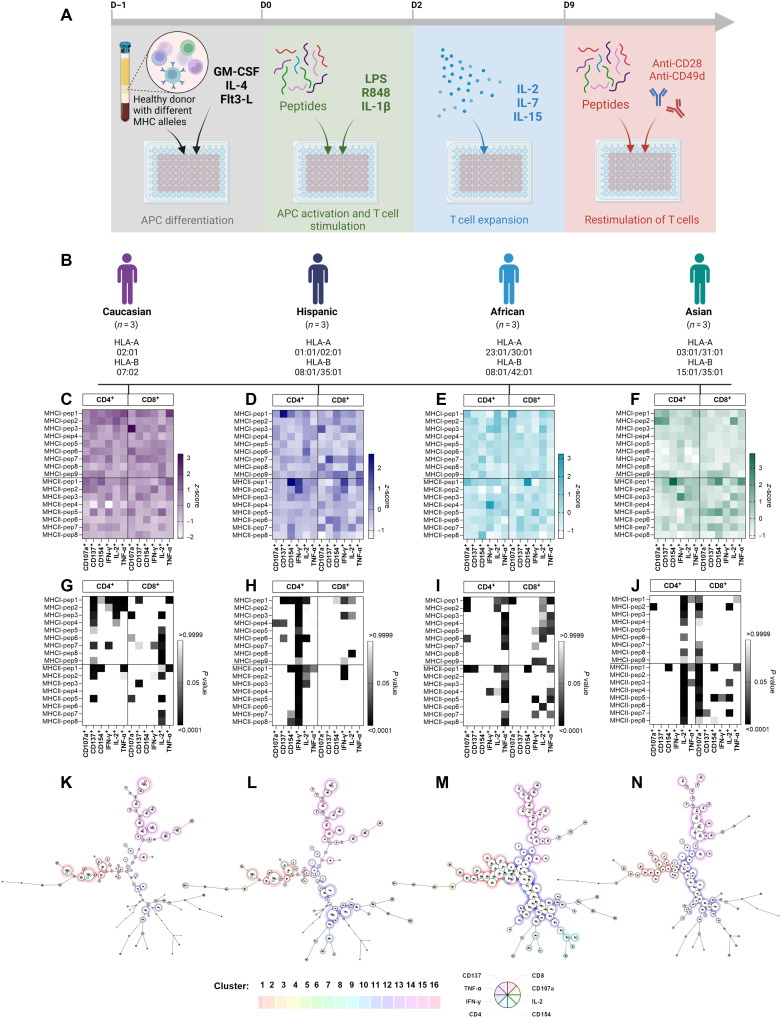
Immunogenic profiles of HLA-typed PBMCs from healthy donors after in vitro expansion with pan-alphaviruses peptides. (**A**) Schematic design of peptide-specific in vitro T cell differentiation and clonal expansion protocol described in (*28*). (**B**) Donors’ descriptives by ethnic group, separated in Caucasian, Hispanic, African, and Asian, respectively. The following plots represent the *z*-score (**C** to **F**) and *P* value (**G** to **J**) of stimulated T cells by ethnic group. (**K** to **N**) Clustering tree of stimulated T cells. External colors identify different clusters, internal colors identify stimulated T cell and activation biomarkers on each cluster, and circle size is related to the yield of stimulated cells. GM-CSF, granulocyte-macrophage colony-stimulating factor; LPS, lipopolysaccharide. APC, antigen presenting cell.

One of the main focuses of the pipeline selection was to tailor the vaccine candidate to the realities where the infectious agent circulates. Because of that, we included the most frequent HLA alleles observed in South America as a pipeline constraint, as described by MD. To evaluate the overall ability of the vaccine candidate peptides to induce T cell responses and the effect of allele variability, we obtained HLA-typed PBMCs [cryopreserved PBMC (ePBMC); ImmunoSpot, USA], selecting the most common HLA alleles on the ethnic groups Caucasian, Hispanic, African, and Asian. Donors’ descriptives can be found in table S6. Regarding surface markers, we included CD107a as a degranulation marker associated with natural killer (NK) cell functional activity. Also, CD137 is a costimulatory protein member of the TNFR family that promotes the proliferation and survival of activated T cells. CD154 (or CD40L), another member of the TNFR family, can correlate with T cell activation, mainly expressed in activated CD4^+^ cells (Fig. 5 and figs. S7 and S8).

To assess the potential immunogenicity of 17 peptides progressing through our vaccine candidate pipeline, we induced T cell–specific responses against each peptide using an immunogenicity assay designed to rapidly prime naïve T cells (Fig. 5A) (*28*). Overall, our data show that several peptides have well-defined immunogenic signatures, leading toward a strong IFN-γ response and moderate IL-2 secretion, with some effectively triggering TNF-α expression (Fig. 5). We expected peptides to be more stimulating in Hispanic and African donors concerning cytokines induction (Fig. 5, D/H and E/I, respectively), due to the inclusion of HLA alleles present in the South American population previously in the pipeline. Both sets of peptides (MHC-I and MHC-II) stimulate strong T CD4–driven IFN-γ response in Hispanic donors, and a T CD8^+^ cell IFN-γ response is rarely observed in PBMCs stimulated by MHC-II–targeted peptides. African donors have well-balanced responses among the evaluated biomarkers, indicating that most peptides could be selected. A strong TNF-α response elicited by stimulated African PBMCs was not associated with an increased expression of surface markers from the same protein family (CD154 and CD137). In general, Caucasian donors promote a milder response regarding cytokine expression and activation markers in comparison to Hispanics, but the responsiveness of MHC-I Pep1 and MHC-II Pep1 is sustained between most of receptors and cytokines tested. Unexpectedly, stimulated Asian PBMCs mainly elicited an IL-2–driven response by both sets of epitopes analyzed.

When analyzing the pattern of cellular response by ethnic group, most of cells cluster similarly among groups but with different biomarker intensities (Fig. 5, L to O). In this analysis, it is clear how the response is milder throughout all cell populations analyzed on Caucasian donors, a clear opposite to the African response. Last, as observed on the murine PBMC, peptide immunogenicity in humans presents different patterns when analyzed by viruses (fig. S9B) and by ethnicity (fig. S9C). These data show that the host immune response to these peptides varies broadly not only by viral complex but also by ethnicity, which illustrates a core challenge of producing a peptide vaccine with broad viral and ethnic coverage. Total stimulated cell percentages are listed in figs. S7 and S8, including experimental controls.

### Immunologic profile of activated T cells on humans preexposed to alphaviruses corroborate in silico and in vitro analysis

**Fig. 6.**
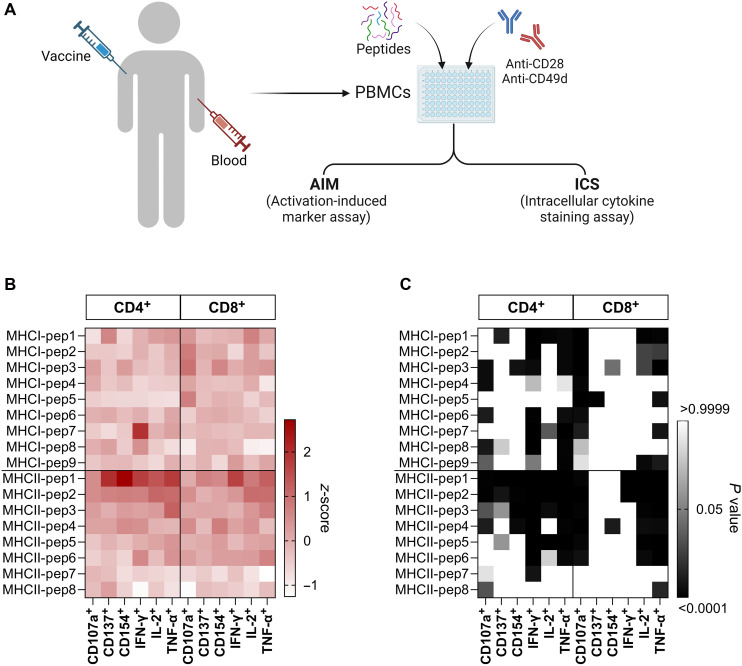
Immunologic profile of activated T cells on human PBMCs followed by in vitro stimulation with alphavirus-specific peptides. PBMCs were collected from donors previously exposed to alphaviruses vaccines TC-83, TSI-GSD 104/inactivated PE-6 strain, and/or TSI-GSD 210/inactivated CM-4884 strain. (**A**) Experimental design of PBMC stimulation. Isolated PBMCs were restimulated in vitro and evaluated by flow cytometry for T cell activation (CD4^+^/CD8^+^) by surface markers (CD107a, CD137, and CD154) and secretion of cytokines (IFN-γ, TNF-α, and IL-2). (**B**) Relative changes (*z*-score) of surface markers and cytokines on preexposed PBMCs after restimulation with peptides. (**C**) Heatmap with the representation of *P* values obtained by two-way ANOVA statistical analysis of (B).

Considering the difficulty of obtaining PBMCs from patients naturally infected with different alphaviruses to corroborate the data shown here, we opt to validate our selected peptides on donors preexposed to candidate alphavirus vaccines. Donors were vaccinated with one or all of the following: TC-83 (VEEV), TSI-GSD 104/inactivated PE-6 strain (EEEV), and TSI-GSD 210/inactivated CM-4884 strain (WEEV) (table S7). Obtained PBMCs were restimulated in vitro as described before. Here, we observed the same pattern of cytokine secretion shown in the previous results. Similar to mouse and naïve primed T cells (Fig. 6A), our peptides elicit a strong IFN-γ, IL-2, and TNF-α responses on PBMCs preexposed to alphaviruses antigens, with MHC-I Pep1, MHC-I Pep3, MHC-I Pep7, MHC-II Pep1, MHC-II Pep2, and MHC-II Pep4 eliciting the most comprehensive T cell activation response (Fig. 6, B and C). Although some donors received TC-83 only or more vaccines, the immunogenicity of the peptides did not present significant changes when analyzed by vaccine types (figs. S10 and S11). Peptide MHC-II Pep1 did not present a good response against mouse PBMCs, which agrees with the pipeline classification as human top-ranked and mouse bottom-ranked (table S3). However, this is the only peptide in a B cell cluster that also activates T cells in any human in vitro validation.

Last, all in silico and in vitro data were included in the final run of the pipeline for the selection of candidates using a design containing cleavable spacers between target pan-alpha epitopes. All selected peptides are now being tested in an in vivo model with the same alphaviruses used for in vitro validation. Given the complexity of B cell response assessment, a prime-boost immunization protocol is in progress, and a preclinical vaccine evaluation is ongoing. To overcome the limitations of in vivo experimental models, the pipeline can also be used to select species-specific immunogens, HLA-target groups, or predicted biochemical properties that affect formulation. In this sense, we selected different final candidates by target species.

## DISCUSSION

The literature on alphavirus vaccines goes back to the 1970s. Several approaches were tested, with different degrees of success. Live-attenuated candidates were developed by introducing mutations or deletions in structural and nonstructural proteins (*29*–*36*), while infectious virions were chemically treated to generate inactivated candidates (*37*–*40*). Formalin-inactivated vaccines require multiple boosts to achieve and maintain detectable neutralizing antibody titers in human recipients and provide incomplete protection against aerosol challenges in animal models (*41*). Attenuated mutants derived from these inactivated vaccine strains showed improved mucosal protection (*42*), but studies described the potential for virulence reversion (*43*). Some candidates, such as the TC-83 VEEV vaccine, have received the Investigational New Drug status, but its high reactogenicity restricted the usage to at-risk personnel (*37*). Other studies pointed out the impact of cross-reactivity among immune responses from different vaccines. Literature describes the potential for immune interference when different vaccines against VEEV, EEEV, and WEEV are subsequently administrated (*41*, *44*, *45*). Pittman and collaborators indicate that vaccinating humans against EEEV and WEEV with inactivated vaccines may interfere with subsequent neutralizing antibody response to the live attenuated VEEV (*44*). This evidence indicates the necessity of developing a multivalent and protective candidate that surpasses individual vaccines’ immune response.

Most of the candidates described in literature are based on the structural protein C-E3-E2-6K-E1 complex. The FDA-approved CHIKV candidates, IXCHIQ and VIMKUNYA, are a live attenuated with a deletion on the nsP3 gene and can generate high levels of neutralizing antibodies (*46*) and virus-like particle vaccines (*47*), respectively. However, IXCHIQ had its biologics license recently suspended by FDA based on serious safety concerns (*48*). Other candidates in clinical phases also contain genes that correspond to structural proteins. Our pipeline ideally identified the most suitable epitopes among proteins E1 and E2 for a broad pan-alpha response. However, the list of most reactive overlapping epitopes also includes peptides from E3, nsp2, nsp3, and nsp4 (Fig. 2 and table S3). Previous work corroborates our findings with noninfectious viral-like particle vaccines and viral-vectored candidates that have shown high immunogenicity against structural proteins (*36*, *49*–*55*) but highlights the necessity of suitable vaccine vectors for a boost on the immune cellular response (*56*).

Although several candidates now in clinical trials are live-attenuated candidates, it is essential to stress the possibility of risk of reversion to a pathogenic virus, which can limit the broad application of this immunogen. Subunit vaccines present exceptional safety and simplified production process, where the immunoinformatic approach can determine potential bottlenecks and optimize a new candidate (*57*). This is especially important in the context population variable genetic pool and high numbers of immunosuppressed individuals, infants, and the elderly (*58*). Thus, strategies that increase the capacity and coverage of subunit vaccines are necessary. Studies aim to compensate for the population’s genetic variation and improve vaccine coverage even for pathogens with well-established immunogens, which is the case of tuberculosis (*59*). Therefore, our pipeline included the genetic background of the population from South America, which has the highest risk of infection to cocirculating alphaviruses, as a constraint for the epitope selection. Our results show that Hispanic and African donors presented more robust activation of T cells compared to Caucasian donors, which can be explained by the most frequent HLA haplotypes observed in South America. Additionally, Khor and collaborators have shown an association between HLA and antibody response after vaccination against SARS-CoV-2 (*60*). In another SARS-CoV-2 vaccine evaluation, Bertinetto *et al.* (*61*) discuss that HLA significantly influences cellular and humoral responses. The difference between the epitope immune profiling in different ethnic groups opens the discussion to the necessity of tuning vaccine candidates to the targeted population. Critically, while our data provide the proof of concept for evidence of allele-specific or population-level differences among ethnic groups, we recognize that the number of available donors per group was limited, and, thus, definite evidence will require substantial investment in the development phase to support evidence of allele-specific or population-level differences.

While most of licensed vaccines are thought to confer protection primarily through the induction of neutralizing antibodies, an increasing body of evidence highlights the underappreciated but critical role of T cells in protective immunity as they contribute to infection control, reduce disease severity, and promote long-term immune memory, even in the face of waning or variant-evaded antibody responses, as reviewed by Sette and Saphire (*62*). In the case of CHIKV, T cells have been implicated in both protective and pathogenic roles, and recent epitope mapping studies have begun to define the landscape of CHIKV-specific T cell targets in humans (*63*). These insights underscore the importance of incorporating T cell–based analyses in vaccine development and immune monitoring pipelines, particularly for diseases where antibody responses alone may not fully capture the complexity of protective immunity.

Bioinformatic tools improve biosafety and coverage of new immunogens. Computational approaches can predict B or T cell–specific epitopes with potential long-lasting protective immunity. As we stated in the introduction, although the development of fully in silico–designed multiepitope vaccines advancing to late-stage clinical evaluation remains relatively recent, a limited number of vaccine candidates are now in clinical trials*.* Although the definition of correlates of protection can be complex for a multitarget vaccine, recent vaccine candidates tend to prioritize the generation of cellular over humoral responses (*64*). However, B cell epitopes and antibody response are well established in the literature, and the lack of knowledge of T cell epitopes or overall cellular response during infection can represent a challenge for a comprehensive vaccine design (*65*). Most of the epitopes selected by our pipeline stimulated T cells and promoted cytokine secretion on at least one of the viruses or species tested in this work. Although selected as MHC-I– or MHC-II–specific epitopes, all the analyses were realized on PBMC pools, and no depletion of T cell populations was performed. It could explain the dual activation of CD4^+^ or CD8^+^ cells by some epitopes, with secretion of IFN-γ, TNF-α, and IL-2. The analysis did not include other important leukocyte populations, like NK cells. In a trivalent modified vaccinia Ankara (MVA)-based vaccine against VEEV, EEEV, and WEEV (*55*), E2 peptides were responsible for a significant IFN-γ response without detectable immune interference seen in terms of neutralizing antibodies, which corroborates peptides MHCII-pep1 and MHCII-pep2 high cytokine secretion and highlights these epitopes as potential candidates. CD107a is commonly used as a surrogate marker for cell degranulation and cytotoxicity, and thoughtful consideration in selecting epitopes that exacerbate its secretion is advised. However, recently, researchers demonstrated that NK cells can play a role in regulating vaccine-elicited T cell and B cell response (*66*). Upon vaccination with a SIV DNA/adenovirus in primates, authors observed a trend of CD107a increasing expression in NK cells stimulated in vitro, suggesting the development of memory-like NK cells and enhanced cytokine response (*67*). Other studies hypothesize that NK cells can modulate the quality of the T and B cell memory responses (*68*). Similar peptide immunogenicity patterns were observed when comparing in vitro the naïve T cell primed to the expanded cells, which means that this approach can be used to prospect new reactive epitopes, even if biological material from infected patients is not available or are restricted to biosafety level 3 or 4.

Beyond alphaviruses, this work represents an iterative approach to vaccine design that serves the unique needs of emerging infectious disease biosurveillance (Fig. 1). These needs are that the workflow from identifying a target pathogen to designing a vaccine candidate is succinct and can be executed quickly. Related to this, such a workflow also needs to be easily reexecuted as more pathogenic data are collected (e.g., new biospecimens being sequenced). Such a workflow needs to serve the needs of ad hoc and exploratory analysis by storing its data in such a way that these analyses can take place on a central data source. Last, such a workflow needs to be constructed transparently and portable as emerging vaccine designs will take place in uniquely distributed settings with many different laboratories and researchers contributing data, performing validation assays, or conducting additional analyses. Moreover, this pipeline can be executed against any collection of proteomes and implements standard workflows such as epitope identification and structural analysis. Moreover, this pipeline (Fig. 1) has a runtime of approximately 48 hours on 256 compute cores, 1000 GB of RAM, and 4x Nvidia Tesla A100 80GB GPUs. Although we used computational resources available at local HPC clusters, the pipeline is designed to be portable to any HPC environment or to distributed cloud computing environments that are commercially available. Our pipeline is entirely coordinated by Nextflow with containerization of all workflow steps. No single step requires more than a single GPU and can be executed with as little as 32 compute cores and 128 GB of RAM, making this pipeline scalable through cloud pipeline engines such as AWS Batch and Azure Batch. We are sanguine that the pipeline presented herein can serve as a repeatable workflow for the iterative design of vaccines against emerging infections and that the broader community can use and even extend this workflow in accordance with the architecture described above.

## MATERIALS AND METHODS

### Experimental design

We developed a pipeline to select T cell immunogenic epitopes from viral proteomes for a multivalent vaccine candidate. Our pipeline considers population coverage, including HLA allele frequency across ethnicities and geographies, as well as murine and primate HLA alleles relevant for preclinical testing. Additional filters include antigenicity, binding affinity, solubility, and stability. All constraints are combined in a score after a series of in silico evaluations. A total of 726 top and bottom-ranked epitopes were selected for initial screening via peptide microarrays using pooled sera from arbovirus-infected humans (*n* = 27), mice (*n* = 19), and NHPs (*n* = 5).

Overlapping peptides were then grouped by binding affinity to MHC-I or MHC-II receptors. Molecular modeling and dynamics simulations confirmed peptide binding and structural stability. T cell immunogenicity was then validated in vitro. First, mice were infected with MAYV or TC-83 (10 per group, in duplicate), and PBMCs were stimulated with selected peptides. Reactivity was assessed by activation marker expression and cytokine production. Second, PBMCs from healthy human donors of various ethnicities (*n* = 4 per group, in duplicate) were used to generate antigen-specific T cells, followed by peptide stimulation and cytokine detection via flow cytometry. Last, PBMCs from vaccinated, alphavirus-exposed donors were similarly tested to confirm immunogenicity.

### Ethics statement

All animal procedures followed University of Texas Medical Branch (UTMB) Institutional Animal Care and Use Committee guidelines (protocol no. 2007080, approved 8 July 2020). Human HLA-typed ePBMCs were obtained from ImmunoSpot (OH, USA), isolated from leukopaks collected via leukapheresis (COBE Spectra/Optia) from Institutional Review Board (IRB)–consented healthy donors at FDA-registered centers, and frozen in CTL-CryoABC medium. Deidentified PBMCs, serum, and clinical data from vaccinated individuals were provided by the UTMB Biorepository for Severe Emerging Infections (BSEI). Samples were collected under IRB-approved protocols at the University of Nebraska Medical Center (no. 0410-23-EP, approved 17 August 2023) and UTMB (no. 23-049, approved 20 October 2023), led by C. Levine.

### Pipeline development

We first collected 54 proteomes of sequenced alphaviruses obtained through NCBI GenBank. These samples were taken from various viral clades as shown in table S1. This process would mimic the identification and submission of newly sequenced viral samples that can be computationally converted into proteomes and added to an expanding biosurveillance dataset. We next developed a pipeline to render vaccine candidates from these submitted proteomes (Fig. 1).

#### Preliminary epitope identification

The pipeline initiates with a concatenation of the collected proteomes into a multi-FASTA file and submission of this file to several specific epitope identification tools. For linear epitope selection, EpiDope is used to detect linear B cell epitopes (*69*), NetMHCIPAN is used to detect linear MHC-I epitopes (*27*), and NetMHCIIPAN is used to detect linear MHC-II epitopes (*27*). For discontinuous B cell epitopes, the pipeline first calculates folded protein structures using ESMFold2 (*70*) and then submits these to DiscoTope (*71*).

#### Secondary epitope evaluation

For MHC-I and MHC-II epitopes, the pipeline takes the highest-ranked epitopes (as ranked by their NETMHCPAN calculated ligand elution) and performs docking simulations with MHC and T cell receptor (TCR) proteins. These epitopes are then used in TCRpMHC models (*72*) to create a protein complex encompassing the peptide epitope, the most predominant MHC-I or MHC-II allele and TCRα and TCRβ chains. This complex is then assessed using the PDB Proteins, Interfaces, Structures, and Assemblies (ePISA) service, after which the epitope-MHC and epitope-TCR interfaces are excerpted, and their free-solvation energy is normalized as the number of standard deviations from the mean across all scored epitopes. This number is then used as a “score” for the stability of the TCR-peptide-MHC complex, and this is recorded in a table for inclusion in the data warehouse.

For B cell epitopes, the pipeline first filters scored epitopes on the basis of whether their originating protein is within a list of target B cell–relevant proteins. This then results in a subselection of scored B cell epitopes that are within this known set of target proteins.

#### Calculation of MHC allele frequencies

Simultaneously with the initial epitope calculation, the pipeline also computes a regional allele frequency table both for inclusion in the data warehouse (see Supplementary Methods 2.1) and for later calculation of vaccine designs (discussed subsequently). The pipeline comes packaged with an extract of the Allele Frequency Net Database (allelefrequencies.net), which accepts a parameter containing the region or regions wherein the vaccine is intended to be used. On the basis of this selection, each extracted subpopulation dataset is searched for regions that match the specified target regions. Then, all matching subpopulations are pooled, and HLA allele frequencies are recalculated on the basis of this new pooled population and included in the data warehouse.

#### Final vaccine payload evaluation

Epitopes that have been highly scored (in the top 10th percentile of average binding affinity across HLA alleles via NetMHCPanI and NetMHCPanII, respectively) are used to assemble full candidates. These candidates are designed via linear optimization using the JessEV epitope selection tool with the Gurobi optimizer, combining five epitopes and associated linkers to preserve epitope identity and maximal peptide representation. Input peptides are trimmed to nine amino acids before submission to JessEV and run for 10 iterations to optimize vaccine candidates. Last, we reconstruct “full-length” versions by replacing the nine–amino acid epitopes in the constructs with their corresponding full-length peptides. Note that, for MHC-I epitopes, no replacement is necessary as they are already nine amino acids in length. For MHC-II epitopes, these are 15 amino acids in length and are truncated by trimming 3 amino acids from each end (6 amino acids in total) before evaluation via JessEV. Last, the selected peptides are then repartitioned and independently affixed to gold nanoparticles to construct a final vaccine candidate ready for use. A complete description of the pipeline architecture can be found in Supplementary Methods 2.2.

### In vitro validation

#### In vitro assessment of pan-alphavirus epitope repertoire using peptide microarray

Evaluation of the pan-alphavirus–restricted peptide repertoire was performed using PEPperCHIP (PEPperPRINT, Heidelberg, Germany) custom peptide microarray. All predicted peptides were included in this microarray, including T cell overlapping epitopes and nonoverlapping epitopes among different alphaviruses (control). For the microarray, 726 peptides were adsorbed in duplicate on the inside of spots located on a microarray glass slide (75.4 mm by 25.0 mm by 1 mm). Sera banks are described in Supplementary Methods 1.1 and table S2. Each slide consisted of five independent microarrays, which were later tested against different sera pools. Each pooled sera were tested in duplicates. Staining protocols were performed according to manufacturer’s instructions. Detailed protocol described on Supplementary Methods 1.2. The PEPperCHIP microarray slide was digitized on the Typhoon Trio (GE Healthcare, IL, USA). Peptide microarray fluorescent signal data were quantified using MAPIX Analyzer software (Innopsys, Carbonne, FR). Results were expressed in fluorescence intensity (median florescence within replicate spots with background subtraction). Graphs were plotted using GraphPad Prism, v10.2.1 (GraphPad Software, Inc., MA, USA).

### In silico validation

#### Molecular modeling

The modeling of the identified MHC-I or MHC-II peptides was conducted using a customized version of AF2M (*25*) (model version 2.3.2, available at https://github.com/google-deepmind/alphafold) and local ColabFold (*26*) (model version 1.5.1, available at https://github.com/YoshitakaMo/localcolabfold/). Consistent with previous studies (*73*), we focused solely on modeling the peptide interaction MHC domain.

For the modeling with AF2M, we used a custom sequence dataset comprising TCR and MHC sequences, similar to (*73*), to speed up the MSA generation step (the customization comprises Uniref90, mgnify, seqres, small bfd, and Uniprot AlphaFold datasets). AF2M was run in multimer mode without template data cutoff, allowing structure relaxation with the Amber force field. We generated five models per target peptide complex, and only the top-ranked model was considered, maintaining all other parameters as default. Modeling using local ColabFold used the alphafold2_multimer_v3 model with 20 recycles. Modeled structures were permitted to relax, while all other ColabFold parameters remained default. To assess the quality of the generated models, we obtained the confidence scores from AlphaFold-based approaches. The multimeric confidence scores represent a combination of predicted template modelling (pTM) and interface predicted template modeling score (ipTM) scores (0.8*ipTM + 0.2*pTM), where higher scores indicate a more accurate modeled binding pose (*25*).

Furthermore, to evaluate whether the modeled peptides bind to the MHC in a manner consistent with known peptide:MHC complexes, thus enhancing our confidence in the model, we compared the backbone positions of modeled peptides with those of peptides bound to MHC in solved 3D structures. This comparison facilitated the identification of modeled peptides with incorrect conformation and binding modes. To conduct this evaluation, we assembled a specific dataset of structures containing MHC-I allele HLA A*02 and MHC-II molecules (in this case, we did not select a specific allele because the number of available MHC-II structures is limited). The dataset only contains peptides of the same length as the identified peptides (9 mers for MHC-I and 15 mers for MHC-II). The MHC-I dataset was obtained from TCRModel2 (https://github.com/piercelab/tcrmodel2/tree/main/data/templates), and the MHC-II dataset was obtained from the curated PANDORA dataset (https://github.com/X-lab-3D/PANDORA) (*74*). The MHC of the modeled complexes was then superposed onto each of the structures in the curated dataset, and the peptide backbone RMSD was computed. Structures with the lowest RMSD to modeled peptides are listed in table S1. The selection of the models generated by AF2M or ColabFold for submission to MD simulations was based on the deviation from known bound complexes.

For MHC-II cases, an additional metric used to assess the modeling quality was the prediction of the peptide core binding positions using NetMHCIIpan (*27*). For this, we used the NetMHCIIpan server (version 4.0, available at https://services.healthtech.dtu.dk/services/NetMHCIIpan-4.0/) was used, with the corresponding DRB1*07:01:01:01 allele and default options.

#### MD simulations

The models of the peptides bound to the MHC obtained with the AF-based approaches served as starting points for MD simulations. Initially, each complex was protonated at pH 7.4 using the pdb2pqr30 program (*75*) and PROPKA for titration state determination (*76*). The N and C termini of the MHC structures were capped with NME and ACE residues with the Python PyMOL package. MD simulations were conducted using Amber 20 suite of programs with the ff14SB force field (*77*). Na^+^ and Cl^−^ counter ions were added to the system using tleap to achieve net neutralization, with an excess of salt to reach a concentration of 150 mM NaCl. Each structure was immersed into a truncated octahedral box (15 Å from the solute) filled with TIP3P water. The PBRadii parameter was set to mbondi2. The system was minimized by 2500 steps of steepest descent minimization followed by 2500 steps of conjugate gradient minimization. Equilibration was performed by heating the system from 0 to 298 K over 200 ps under NVT conditions, with protein atom positions restrained. This was followed by density equilibration for 500 ps under NPT conditions without restraints. For each complex, the production run was performed at 298 K under NPT conditions for 2 ns with a time step of 2 fs, in triplicate at least. Temperature was maintained using a Langevin thermostat with a collision frequency of 5 ps^−1^. Hydrogen-containing bonds were constrained using SHAKE (*78*). Long-range electrostatic interactions were calculated using particle Mesh Ewald and short-range nonbonded interactions were calculated with a 9-Å cutoff. The simulations were conducted using the GPU-accelerated PMEMD program, a part of AMBER 20 package.

The MD trajectories were analyzed in R using the Bio3D package (*79*). All analyses were performed after the superposition of trajectory frames by the backbone atoms of the MHC molecules using Bio3D fit.xyz function. The RMSD was calculated using the Bio3D rmsd function.

Through the same MD simulation protocol, simulations of three crystallographic structures of peptide bound to MHC-I (PDB IDs: 7u21, 1duz, and 5e00) were performed to be used as reference for the peptide stability. These structures were selected based on resolution criteria (<2 Å) and absence of crystallographic artifacts. To compare the positions of the peptide anchoring site P2 and PΩ along the trajectories, we used as reference the positions of the anchors from the structure 5hhn with the peptide bound to the MHC-I molecule. Images of the structures were created using ChimeraX program (*80*).

### In vivo validation in mice

#### Peptide synthesis

Custom peptide libraries for MHC-I and MHC-II peptides were chemically synthesized by GenScript (NJ, USA). Each peptide had >95% purity as determined by high performance liquid chromatography. MOG and CEFT peptide pools were commercially available at JPT Peptide Technologies (Berlin, Germany). Each peptide was resuspended in ddH_2_O or dimethyl sulfoxide (DMSO; Sigma-Aldrich, MO, USA), according to the manufacturer’s recommendation. Peptides were used at a final concentration of 1 μM.

#### Mice infection and PBMC isolation

A cohort of 30 6-week-old C57BL/6 mice (the Jackson Laboratory, ME, USA) were divided by mock-infected, MAYV-infected, and TC-83-infected groups (Fig. 4A). Viruses and cell origins are described on Supplementary Methods 1.3. Each group contained five females and five male animals. Animals were injected intraperitoneally with 5.0 log_10_ focus-forming units and weighed daily for a wellness checkup. Blood was collected retro-orbitally at the second day postinfection to assess viremia. On the 14th day postinfection, mice were euthanized for tissue harvest and blood collection by terminal cardiac puncture. Neutralization assay was used to assess specific-antibody secretion after infection. Neutralizing antibodies were quantified by plaque reduction neutralization test (PRNT; Supplementary Methods 1.4).

PBMCs were isolated by density-gradient sedimentation using Ficoll-Paque PREMIUM (density, 1.084 g/ml; Cytiva, MA, USA), according to the manufacturer’ protocol, and cryopreserved in cell recovery medium containing 10% DMSO (Gibco, Thermo Fisher scientific, MA, USA), supplemented with 90% heat-inactivated fetal bovine serum (FBS; HyClone Laboratories, UT, USA), and stored in liquid nitrogen until used in the reactivity assays.

#### Murine T cell reactivity against pan-alphavirus peptides

Peptide T cell immunogenicity was evaluated by flow cytometry. For this, cryopreserved PBMCs from infected animals were thawed with CTL Anti-Aggregate Wash solution (ImmunoSpot, OH, USA), following the manufacturer’s recommended procedure. Cells were counted and viability measured prior incubation of 10^6^ cells per well for 1 hour in a 37°C, 5% CO_2_ incubator in a sera-free medium. After, cells were stimulated ex vivo with individual peptides for reactivity assessment. Briefly, PBMCs were cultivated with a final concentration of 1 μM peptide, while the positive control was a mixture of phorbol ester, phorbol 12-myristate 13-acetate (PMA; 50 ng/mL), and ionomycin (1 μg/ml) and the negative control was DMSO (UT). To all conditions were added a stimulation solution containing anti-CD3/anti-CD28 (2 μg/ml) at the beginning of the treatment. In all stimulation conditions, BD GolgiPlug (11 μl/ml; BD Biosciences, NJ, USA) and BD GolgiStop (11 μl/ml BD Biosciences, NJ, USA) were added for the last 6 to 8 hours culture. Cells were incubated in a 37°C, 5% CO_2_ incubator, following kinetics time points and harvested with 2, 4, 6, 12, and 48 hours after treatment. Each condition was tested in triplicate.

Last, cells were harvested, washed, stained for surface markers, fixed and permeabilized with a Cytofix/Cytoperm Kit (BD Biosciences, NJ, USA), and then stained for specific intracellular molecules (table S8). At least 250,000 singlet events (PBMCs) were acquired, with 50,000 events on the CD3^+^ gate, on a FACS Symphony A5 (BD Biosciences, NJ, USA) and BD LSRFortessa Cell Analyzer (BD Biosciences, NJ, USA) analyzed using FlowJo software, v10 (Treestar Inc., OR, USA). For all samples, gating was established using a combination of isotype and fluorescence-minus-one controls.

### Human T cell immunogenicity evaluation

#### Expansion of human alphavirus-peptide specific T cells from healthy donors

PBMCs were thawed and expanded as previously described (*28*). PBMC donors’ descriptives can be found in table S6. Briefly, cells were plated (day 0) with granulocyte-macrophage colony-stimulating factor, IL-4, and Flt3-L overnight to mature antigen presenting cells. On day 1, lipopolysaccharide, R848, and IL-1b were added with peptides (1 μM each). Starting on day 2 and every 2 to 3 days after that, IL-2, IL-7, and IL-15 were added. On day 9 cells were washed, counted, and replated with anti-CD28, anti-CD49d, and desired peptides for 8 hours before staining for flow cytometry (Fig. 4A). The list of controls includes positive control (PMA, 50 ng/ml; and ionomycin, 1 μg/ml), diluent control (DMSO), and negative control (UT). In all stimulation conditions, BD GolgiPlug (11 μl/ml; BD Biosciences, NJ, USA) and BD GolgiStop (11 μl/ml; BD Biosciences, NJ, USA) were added for the last 6 to 8 hours culture. Each condition was tested in triplicate.

Cells were harvested, washed, stained for surface markers, fixed and permeabilized with a Cytofix/Cytoperm Kit (BD Biosciences, NJ, USA), and then stained for specific intracellular molecules (table S8). At least 250,000 singlet events (PBMCs) were acquired, with 50,000 events on the CD3^+^ gate, on a FACS Symphony A5 (BD Biosciences, NJ, USA) and BD LSRFortessa Cell Analyzer (BD Biosciences, NJ, USA) analyzed using FlowJo software, v10 (Treestar Inc., OR, USA). For all samples, gating was established using a combination of isotype and fluorescence-minus-one controls.

#### Immunogenic profile of human PBMCs preexposed to vaccine antigens following peptide stimulation in vitro

PBMC collection, isolation, and cryopreservation were performed by the UTMB BSEI team (table S7). Briefly, whole blood was collected in a sodium citrate–treated Mononuclear Cell Preparation Tube, and PBMCs were isolated following the manufacture’s protocol. Isolated PBMCs were stored in FBS containing 10% DMSO at −196°C until use. Whole blood was collected in a serum separator vacutainer and allowed to clot before centrifugation. Sera was aliquoted and stored at −80°C until use. In vitro stimulation, cell harvesting, and flow cytometry staining procedure is described in the “Expansion of human alphavirus-peptide specific T cells from healthy donors” section.

### Quantification and statistical analysis

Various statistical methods were used to assess group differences, correlations, and associations. Because of data heterogeneity, most groups were *z*-score standardized. Flow cytometry data were collected using BD FACSDiva Software (BD Biosciences, NJ, USA). Data were processed in FlowJo software, v10.10 (BD Biosciences, NJ, USA), using DownSample, Uniform Manifold Approximation and Projection (UMAP), and FlowSOM plugins. Graphs were created with GraphPad Prism v10.2.1 (GraphPad Software, MA, USA). Statistical tests for in vitro and in vivo experiments included paired *t* test, one-way analysis of variance (ANOVA), and Kruskal-Wallis, as indicated in the figure legends.

For in vitro epitope testing via slide microarray with preexposed sera, background fluorescence was subtracted from 635-nm readings to yield signal per peptide, used in Fig. 2F. Each epitope was BLASTed against viral proteomes and marked as representative if identity was ≥90%. For T cell and PBMC peptide stimulation, PBMCs were tested in triplicate by ethnicity and virus. Cytokine values were normalized to a 0-1 scale by dividing by the highest response across epitopes. The mean and 95% confidence intervals from replicates were used to define error bounds.
