# Supplementary material for: Integrated reiterative pipeline for rapid epitope-based pan-alphavirus vaccines
Source: Sci Adv. 2026 Mar 11;12(11):eaeb2066. doi: 10.1126/sciadv.aeb2066 (PMC12978219; doi:10.1126/sciadv.aeb2066)
Supplement: Supplementary file 1 — Supplementary Methods Figs. S1 to S11 Tables S1 to S8 [file sciadv.aeb2066_sm.pdf]

Science Advances
AAAS

Supplementary Materials for

Integrated reiterative pipeline for rapid epitope-based pan-alphavirus vaccines

Alice F. Versiani *et al.*

Corresponding author: Nikos Vasilakis, nivasila@utmb.edu

Sci. Adv. **12**, eaeb2066 (2026)
DOI: 10.1126/sciadv.aeb2066

This PDF file includes:

Supplementary Methods
Figs. S1 to S11
Tables S1 to S8

1. **Supplementary methods:**

1.1 Peptide microarray sera banks:

Mouse and nonhuman primates (NHP) sera banks were selected from laboratory collection and re-tested by PRNT for confirmation (Table S2). The mouse sera biobank is constituted of 19 samples divided by naïve, VEEV-infected, MAYV-infected, CHIKV-infected, and ZIKV-infected – as an off-target control. NHP sera bank (n=5) only includes CHIKV-infected pool sera, ZIKV-infected sera, and naïve. Animal samples were collected following UTMB policy as approved by the UTMB Institutional Animal Care and Use Committee (IACUC), protocol number 2007080 for mouse models (approved on 07/08/2020), and protocol number 1912100 for NHP models (approved on 12/02/2019).

We obtained 16 pre-characterized CHIKV-positive human serum from a prospective arboviral population cohort maintained in Sao Jose do Rio Preto, Brazil. The current research was conducted in compliance with Resolution 466/12 of the National Health Council of the Ministry of Health of Brazil. The study was conducted according to the guidelines of the Declaration of Helsinki and done in retrospective samples with the consent term approved by the institutional review board (IRB) of the Ethics Committee of the Faculdade de Medicina de São José do Rio Preto (protocol codes 15461513.5.0000.5415, approved on April 7, 2015, and 14262619.0.0000.5415, approved on August 13, 2019). Confidentiality was ensured by anonymizing all samples before data entry and analysis.

Other human samples, including Madariaga virus (MADV, n=4) and Venezuelan equine encephalitis virus (VEEV, n=6), were obtained in collaboration with the Gorgas Memorial Institute of Health Studies in Panama City, Panama. This study adopts a cross-sectional design and involves data collection conducted in October 2018 among individuals residing in the community of Aruza, Darién, Panama (protocol code 117/CBI/ICGES/23, approved on May 10, 2023). Commercially available naïve human sera, certified as reference material (Ref# NIST909C Sigma-Aldrich, MO, USA), was used as control.

1.2 Peptide microarray staining procedure:

The first step of the assay consisted of pre-labeling the microarray glass slide with secondary antibody to exclude background reactivity. Mouse-specific slides were coated with goat anti-mouse IgG/Cy5 (Ref# ab6563, Abcam, MA, USA), human-specific slides were coated with goat anti-human IgG/Cy5 (Ref# ab 97172, Abcam, MA, USA), and nonhuman primate slides were coated with goat anti-monkey IgG (Ref# 617-101-012, Rockland Immunochemicals, PA, USA) custom conjugated with Cy5 by PEPperPRINT (Heidelberg, GER).

After the background staining, blocking and washing steps, the microarray slides were incubated for 16 hours at 2-8°C with pre-validated sera pool from mice, nonhuman primates, and humans infected by different alphaviruses (CHIKV, MAYV, VEEV, and MADV), diluted 1:500 in staining buffer. After wash steps, microarray slide incubated with specific-secondary antibodies (described above) and anti-HA/Cy3 control antibody previously diluted (1:2,000) in staining buffer. Throughout the assay, all incubations were performed under constant agitation in an orbital agitation system (140 rpm).

1.3 Viruses and Cells:

Animals were infected using Mayaro virus (MAYV) CH strain and the Venezuelan equine encephalitis virus (VEEV) TC-83. TC-83 (IAB serotype) was chosen for its use the ABSL2 and increased safety profile compared to other VEEV strains. However, TC-83 were previously used in VEEV pathogenic studies due to its virulence and pathogenicity similar to the original virus *(24)*. Virus and vaccine strains were obtained from the World Reference Center for Emerging Viruses and Arboviruses (WRCEVA) at the University of Texas Medical Branch (Galveston, TX). The viruses were passaged once in Vero cells to generate working stocks.

Viremia and neutralization assays were performed in VERO cell line (ATCC® CCL-81™), maintained with DMEM (Gibco, Thermo Fisher scientific, MA, USA), supplemented with 10% heat-inactivated fetal bovine serum (FBS, R&D Systems, USA) and 1% penicillin-streptomycin solution (10^4 U/ml and 10^4 µg/ml solution, respectively) (PenStrep, Gibco, Thermo Fisher scientific, MA, USA).

1.4 Plaque reduction neutralization test (PRNT):

Briefly, sera were pooled into separate groups (male and female), serially diluted in duplicates, and incubated with 50 PFU of MAYV CH strain or TC-83 vaccine strain (final dilutions 1:20 up to 1:640). After 1h, antibody-virus solution was inoculated onto 12-well plates of Vero cells (DMEM supplemented with 2% FBS and 1% PenStrep), and non-neutralized virus was allowed to infect for one hour in a 37°C, 5% CO2 incubator. Following this incubation, an overlay of Opti-MEM (Gibco, Thermo Fisher scientific, MA, USA) supplemented with 2% FBS, 1% PenStrep, and 1% carboxymethylcellulose (Sigma-Aldrich, MO, USA) was added to the wells and the plates were returned to the 37°C, 5% CO2 incubator. After two days, plates were fixed with 10% buffered formalin and stained with crystal violet. Each dilution was tested in duplicity, and the number of plaque-forming units (PFU) was recorded as the average of the number observed in each test. The $PRNT_{50}$ and $PRNT_{80}$ titer is the highest serum dilution able to neutralize at least 50% or 80%, respectively, of plaque formation when compared to virus-only infected control cells. Prior to testing, all sera were incubated at 56°C for one hour to inactivate complement proteins.

2. Pipeline Development (continued)

The pipeline begins with a list of viral proteins as a multi-FASTA file. From here, vaccine design proceeds through several phases. First, each peptide is evaluated as a potential MHC-I epitope, MHC-II epitope, or B-cell epitope. De-duplication of these peptides is performed using CD-HIT. Additionally, a geographic region (e.g. Brazil) is selected at initiation of the pipeline which is used by the pipeline to select predominant MHC alleles for that region. The proteins corresponding to these MHC alleles are used to simulate epitope/TCR/MHC docking and to score binding affinity and docking stability. Finally, identified MHC-I and MHC-II epitopes are selected and prioritized on the basis of binding affinity as well as presence across human, mouse, and non-human primate samples. Epitopes that pass selection in these steps are then provided to JessEV to perform optimization in selecting the best 5 peptides that cover both MHC-I and MHC-II binding while avoiding T-cell cleavage within epitopes. Finally, selected epitopes are folded to confirm that epitopes remain exposed and selected for inclusion in the nanoparticle vaccine candidate.

All calculated epitopes are transformed into tabular output and saved to disk both for use in subsequent pipeline steps as well as for compilation in a data warehouse for *ad-hoc* and exploratory analyses.

a. Data Warehouse Construction

As mentioned in the Materials and Methods section, each step of the pipeline creates tabular output and retains that output in order to facilitate ad-hoc or exploratory analysis. These assets include the following:

- A table of all input proteins with their accession numbers, host organism, and phylogeny.
- A table of all identified B-cell epitopes (both from EpiDope and Discotope) and their predicted immunogenicity.
- A table linking B-cell epitopes to their associated submitted protein.
- A table of all identified T-cell epitopes (both from NetMHCPan and NetMHCIIPan) including their predicted immunogenicity.
- A table linking T-cell epitopes to their associated submitted protein.
- A table of all TCR-pMHC multimers by MHC and peptide and their corresponding surface area and free solvation energy.

- A table of allele frequencies by selected region.
- A table of all candidates linked to their constituent T-cell epitopes.
- A table of CD-Hit clusters of T-Cell epitopes including cluster assignment, centroid sequence, and centroid epitope ID.
- A table of CD-Hit clusters of input proteins including cluster assignment, centroid sequence, and centroid protein ID.
- A table of submitted microarray results linking T-cell epitopes by epitope ID to measured fluorescence.

b. Pipeline architecture

The pipeline described here relies on NextFlow, which is a workflow orchestration tool popular in bioinformatics. Also, the pipeline implements "containerization" which is a popular design approach that confines each analytical task to a specific, reproducible environment. Popularly, these containers can be created and used on any major operating system and can be controlled using either Docker or Singularity both of which are predominant container management tools. Importantly, NextFlow encourages a specific design pattern which this pipeline embraces. That design pattern can be succinctly summarized as follows:

- Every task has a clearly defined input and output.
- Every task can be made into a container.
- Every task's container recipe is tracked as part of the pipeline's source code, allowing any user to rebuild and reproduce tasks and their aggregate workflows.

This results in the pipeline being represented as a directed acyclic graph (DAG) connecting each analytical operation (corresponding to a container) with intermediating channels that route the outputs of one or more source tasks into the inputs of one or more destination tasks. Importantly, containerization and orchestration via NextFlow also means that this pipeline is highly portable in terms of where computation is performed. This means that vaccine design efforts could implement this on local servers or in any of the main cloud providers (Amazon Web Services, Microsoft Azure, Google Cloud). It is even possible to have certain analytical steps performed in separate environments as a combination of these options. The associated GitHub repository (https://github.com/pmccaffrey6/immunoinformatics_platform) contains the source code for the pipeline itself as well as the source code required to build the task-level containers and the relevant configuration and setup code required to execute the pipeline.

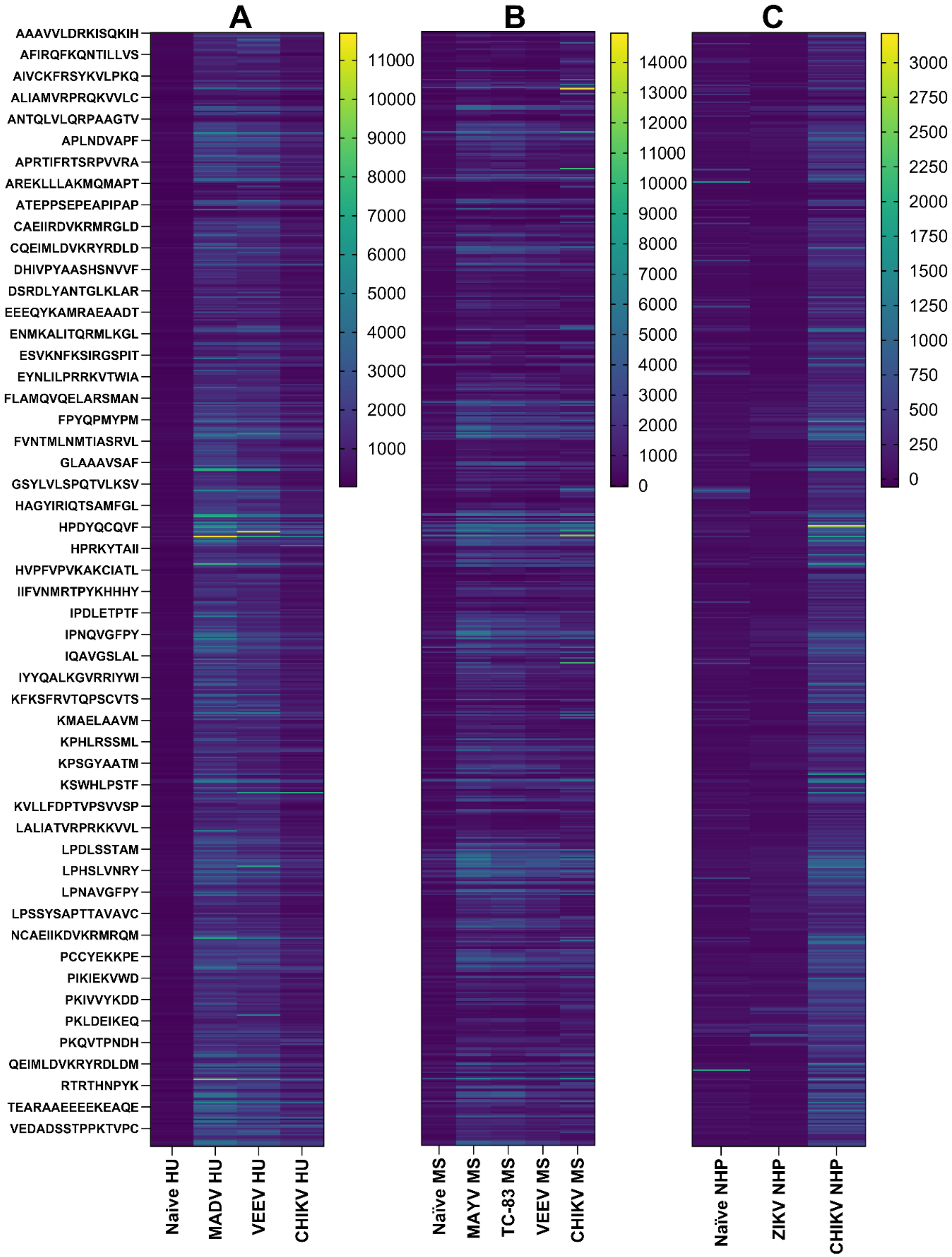

Figure S1. Heatmap analysis of the peptide microarray intensity signal tested against different sera pools. (A) Human sera pools. (B) Mouse sera pools. (C) Non-human primate sera pools. Y-axis indicates peptides, X-axis indicates sera pool tested.

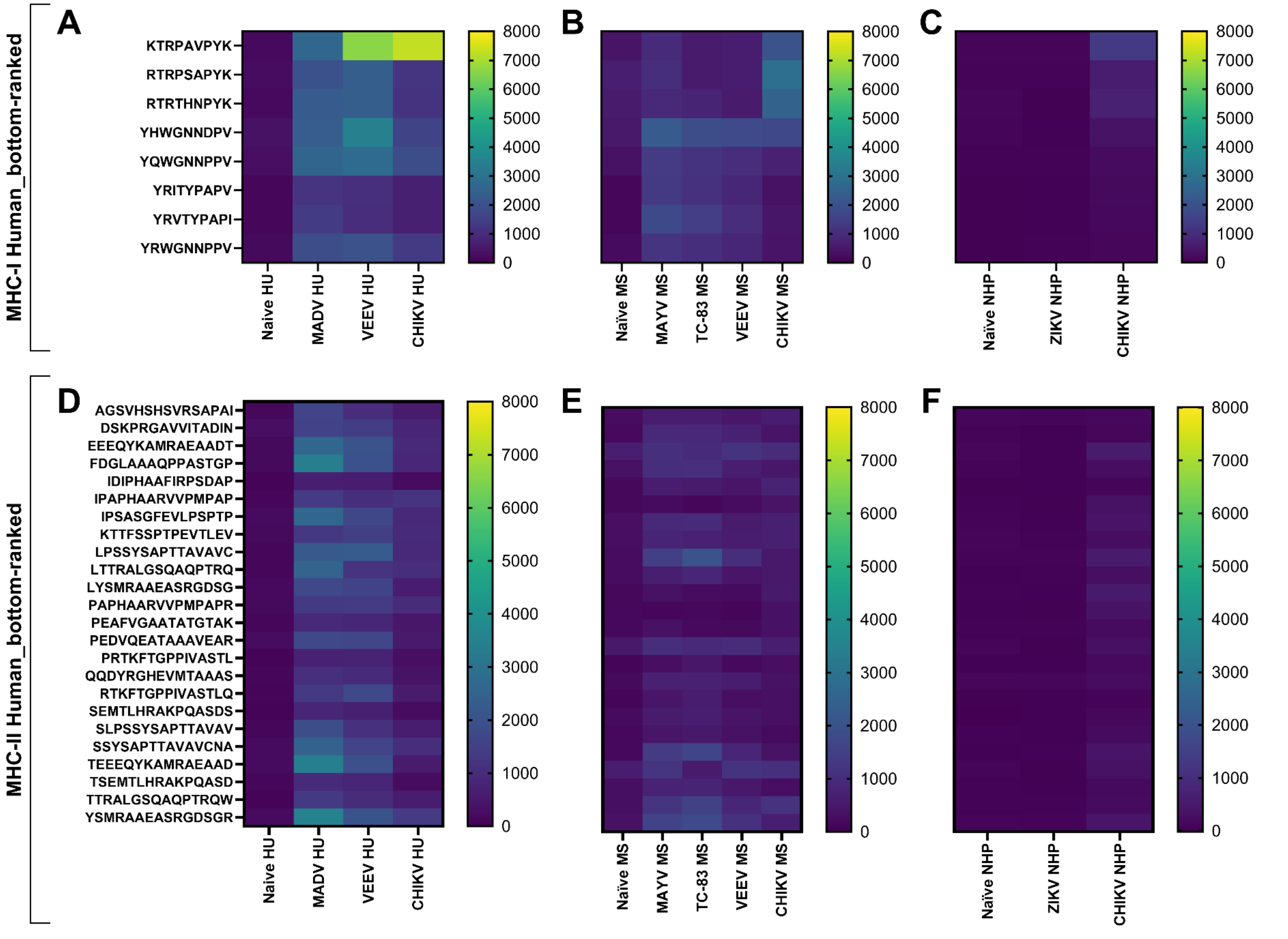

Figure S2. Heatmap analysis of peptide microarray intensity signal of the human bottom-ranked epitopes tested against different sera pools. (A) MHC-I epitopes, human sera pools. (B) MHC-I epitopes, mouse sera pools. (C) MHC-I epitopes, non-human primate sera pools. (D) MHC-I epitopes, human sera pools. (E) MHC-I epitopes, mouse sera pools. (F) MHC-I epitopes, non-human primate sera pools. Y-axis indicates peptides, X-axis indicates sera pool tested.

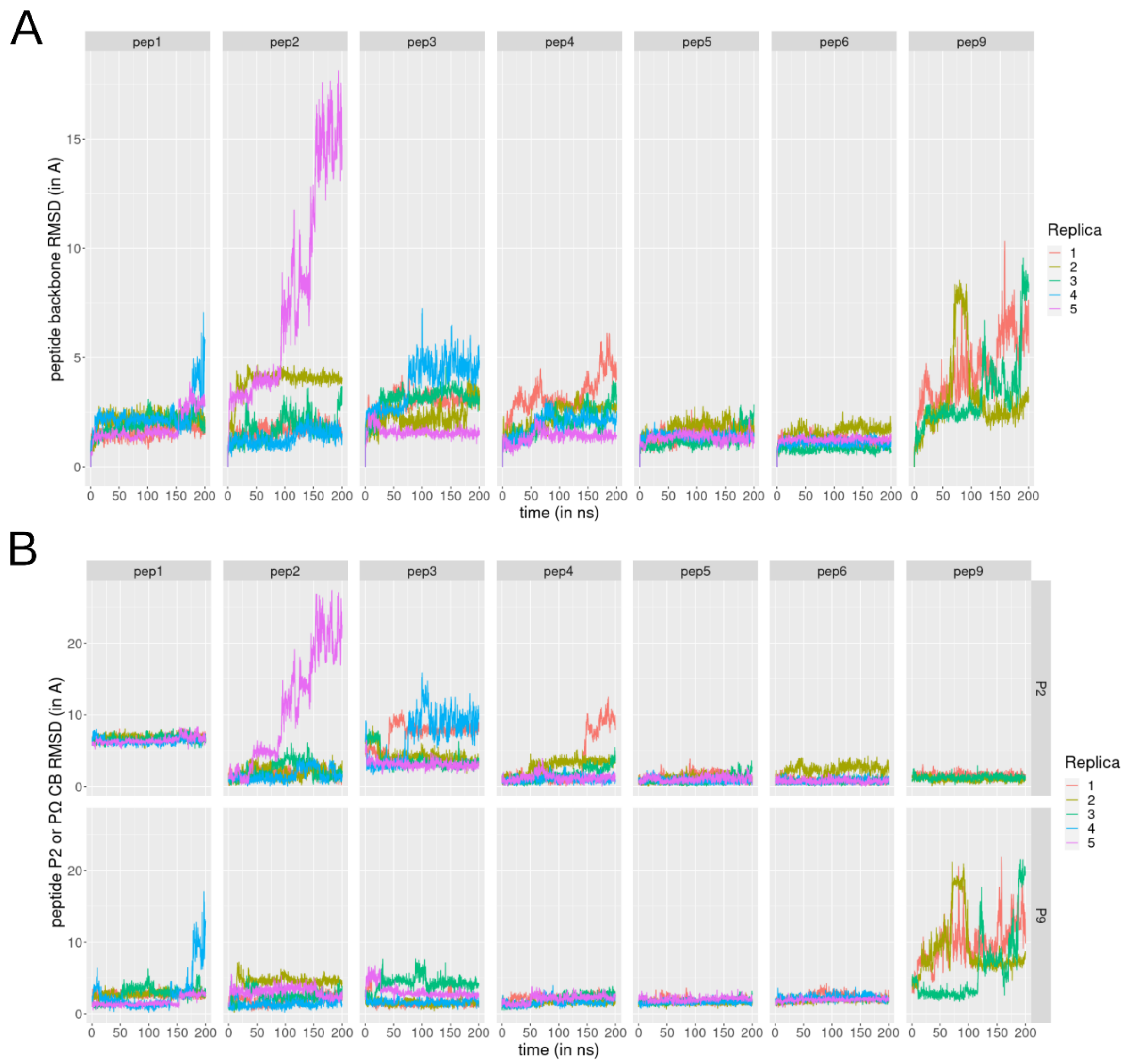

Figure S3. MD simulation analysis of MHC-I bound peptides. (A) Peptide backbone RMSD (in Å) calculated after trajectory superposition by the MHC in five independent replicas. The simulation duration for each replica is 200 nanoseconds, and each line color corresponds to a different replica. (B) Carbon beta (CB) deviation for the peptide positions P2 (upper row) or PΩ (bottom row) in comparison to the same positions in a peptide complex (PDB ID: 5hhn) during the simulations for each peptide.

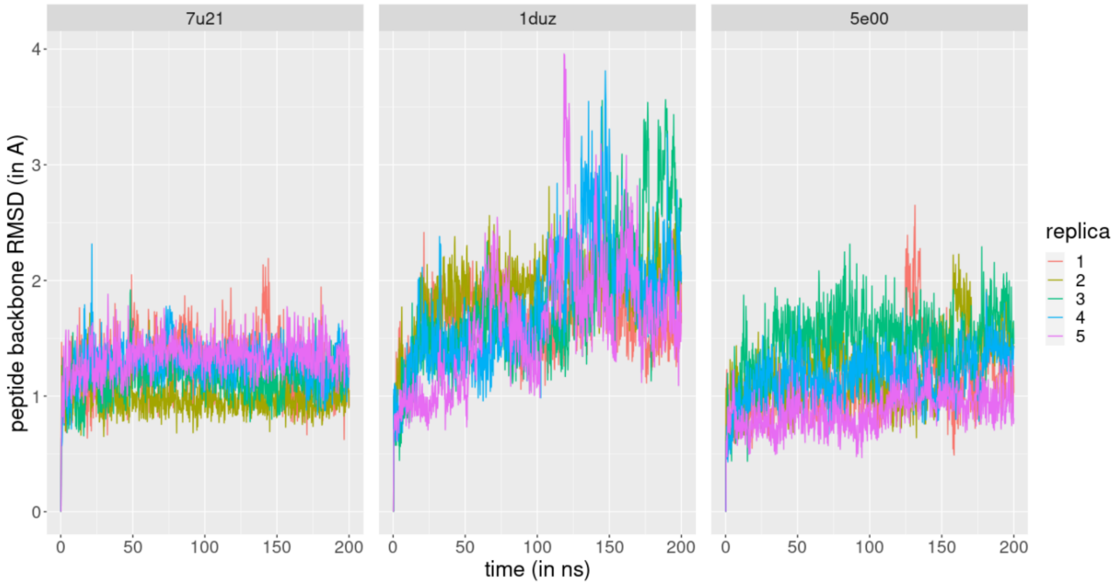

Figure S4. MD simulation analysis of MHC-I bound reference peptides. Peptide backbone RMSD (in Å) calculated after trajectory superposition by the MHC in five independent replicates. The time of simulation is 200 nanoseconds, and each line color corresponds to a different replicate. The reference PDB structures used for the simulations are: 7u21, 1duz and 5e00. The median RMSD of the last half of the simulations considering all replicas is 1.2, 1.9 and 1.2 Å, respectively.

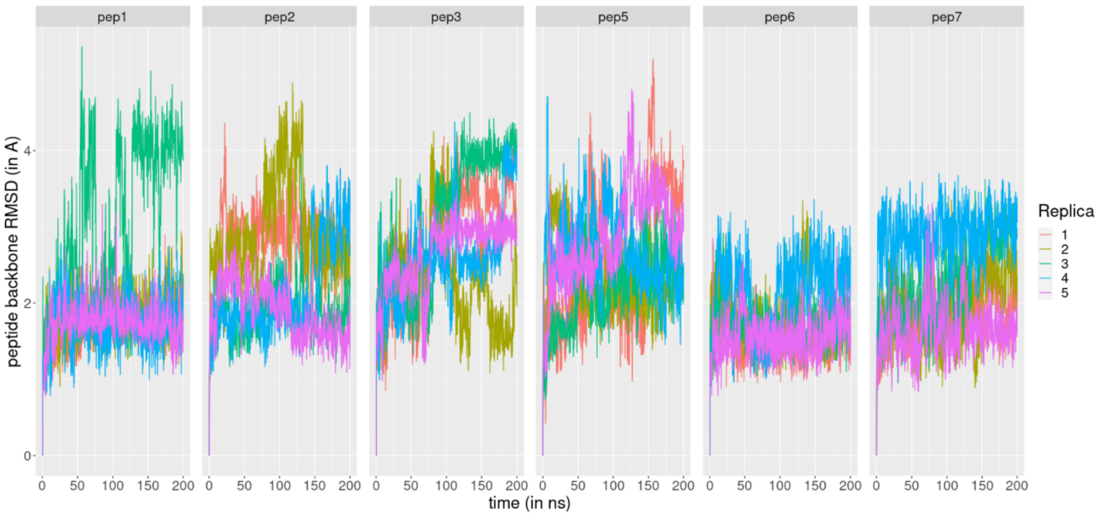

Figure S5. MD simulation analysis of MHC-II bound peptides. (A) Peptide backbone RMSD (in Å) calculated after trajectory superposition by the MHC in five independent replicas. The time of simulation is 200 nanoseconds, and each line color corresponds to a different replicate. Of note the peptide backbone deviation also includes peptide N and C-terminal regions outside the binding score.

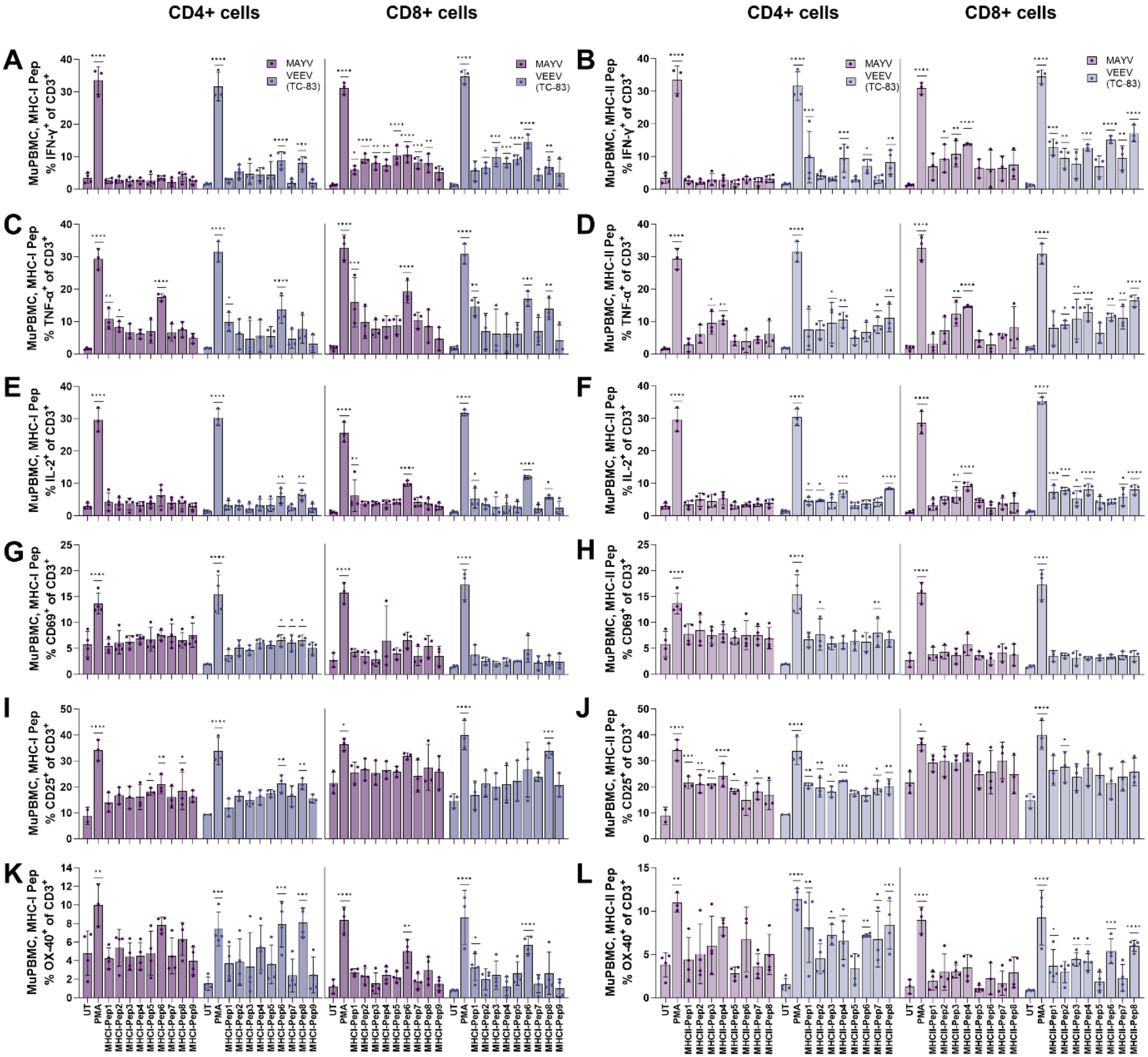

Figure S6. T-cell activation surface markers and cytokine secretion after pre-exposed murine PBMCs are stimulated with pan-alpha peptides. PBMCs from a infected mice were stimulated with the vehicle control (UT, untreated), positive control (PMA), and test alphaviruses peptides. (A) MHC-I peptides, IFN-gamma+ cells. (B) MHC-II peptides, IFN-gamma+ cells. (C) MHC-I peptides, TNF-alpha+ cells. (D) MHC-II peptides, TNF-alpha+ cells. (E) MHC-I peptides, IL-2+ cells. (F) MHC-II peptides, IL-2+ cells. (G) MHC-I peptides, CD69+ cells. (H) MHC-II peptides, CD69+ cells. (I) MHC-I peptides, CD25+ cells. (J) MHC-II peptides, CD25+ cells. (K) MHC-I peptides, OX-40+ cells. (L) MHC-II peptides, OX-40+ cells. Percentage of T-cells was measured by flow cytometry. Data were represented as mean ± standard deviation (SD). p-values obtained by 2-way ANOVA with Dunnett's multiple comparisons test. (*) p<0.5; (**) p<0.01; (***) p>0.001; (****) p<0.0001.

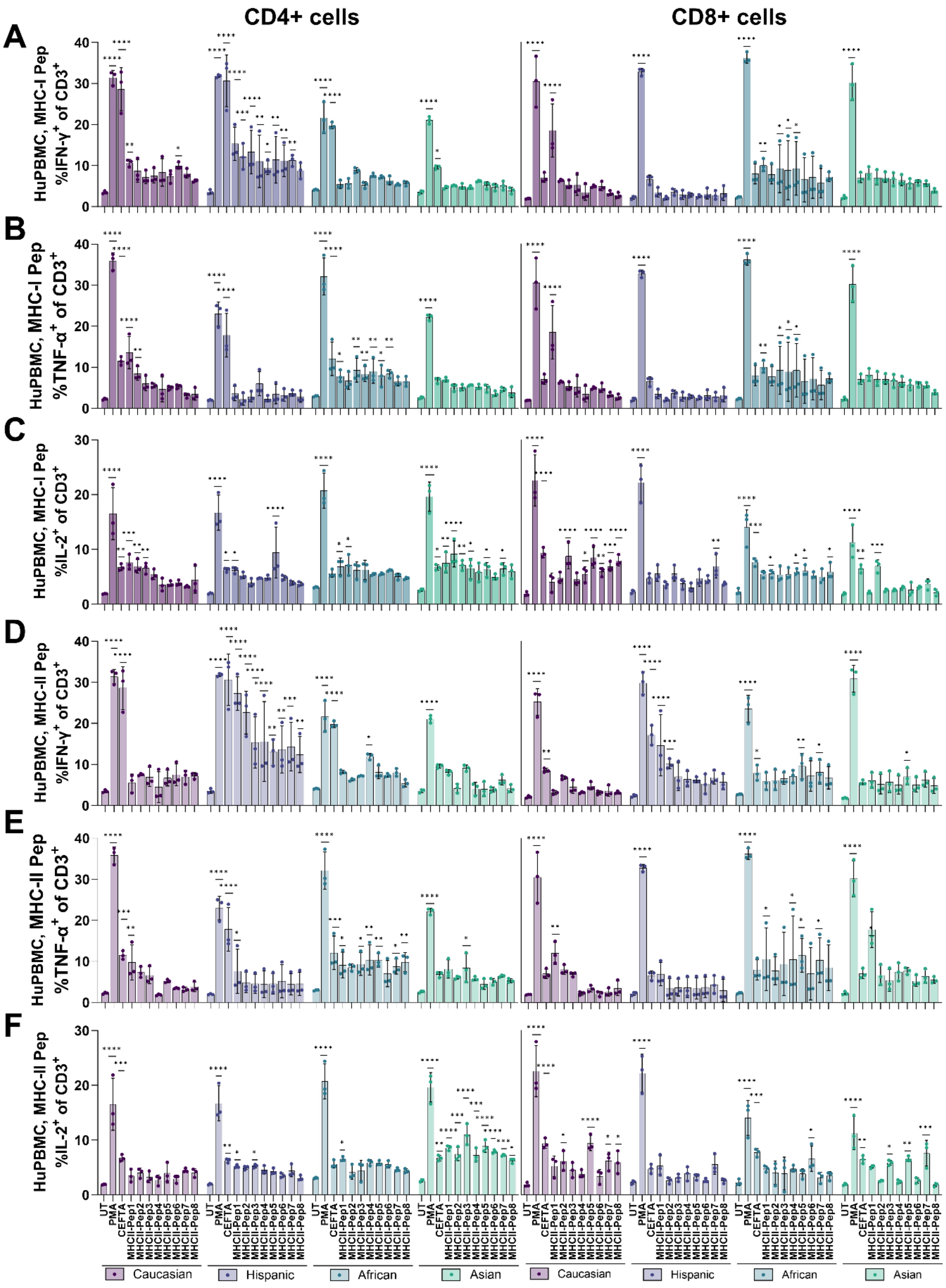

Figure S7. **Cytokine induction on human PBMCs followed by *in vitro* T-cell expansion with alphaviruses peptides.** PBMCs from healthy donors were expanded following stimulation with the vehicle control (UT, untreated), positive control (PMA), CEFT (which contains known viral epitopes), and test alphaviruses peptides. Donors were grouped by ethnicity: Caucasian, Hispanic, African, and Asian. (A) MHC-I peptides, IFN-gamma+ cells. (B) MHC-I peptides, TNF-alpha+ cells. (C) MHC-I peptides, IL-2+ cells. (D) MHC-II peptides, IFN-gamma+ cells. (E) MHC-II peptides, TNF-alpha+ cells. (F) MHC-II peptides, IL-2+ cells. Left graphs indicate CD4+ cells, right graphs indicate CD8+ cells. Percentage of T-cells was measured by flow cytometry. Data were represented as mean ± standard deviation (SD). p-values obtained by 2-way ANOVA with Dunnett's multiple comparisons test. (*) p<0.5; (**) p<0.01; (***) p>0.001; (****) p<0.0001.

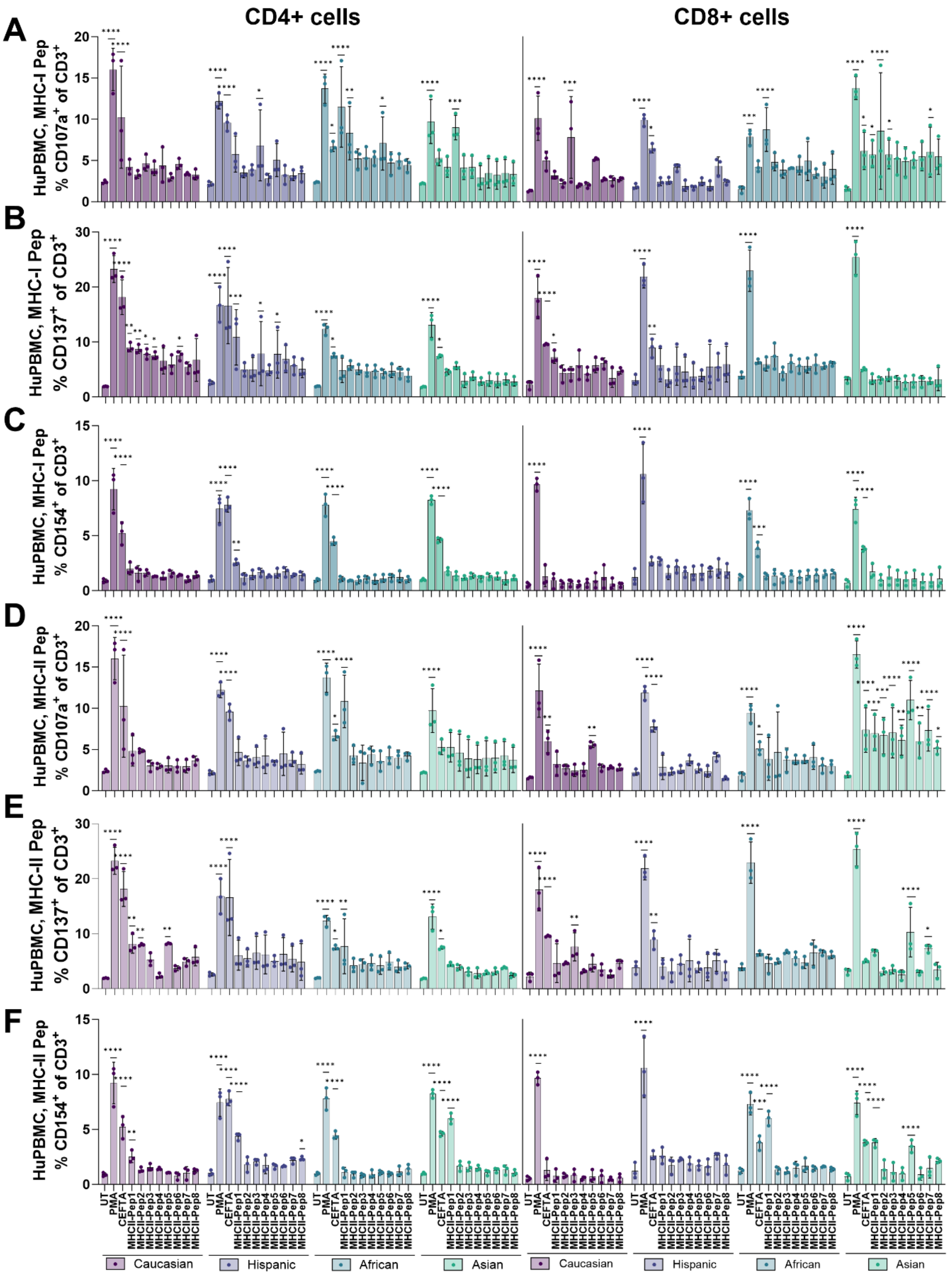

Figure S8. Surface markers induction on human PBMCs followed by *in vitro* T-cell expansion with alphaviruses peptides. PBMCs from healthy donors were expanded following stimulation with the vehicle control (UT, untreated), positive control (PMA), CEFT (which contains known viral epitopes), and test alphaviruses peptides. Donors were grouped by ethnicity: Caucasian, Hispanic, African, and Asian. (A) MHC-I peptides, CD107a+ cells. (B) MHC-I peptides, CD137+ cells. (C) MHC-I peptides, CD154+ cells. (D) MHC-II peptides, CD107a+ cells. (E) MHC-II peptides, CD137+ cells. (F) MHC-II peptides, CD154+ cells. Left graphs indicate CD4 + cells, right graphs indicate CD8+ cells. Percentage of T-cells was measured by flow cytometry. Data were represented as mean ± standard deviation (SD). p-values obtained by 2-way ANOVA with Dunnett's multiple comparisons test. (*) p<0.5; (**) p<0.01; (***) p>0.001; (****) p<0.0001.

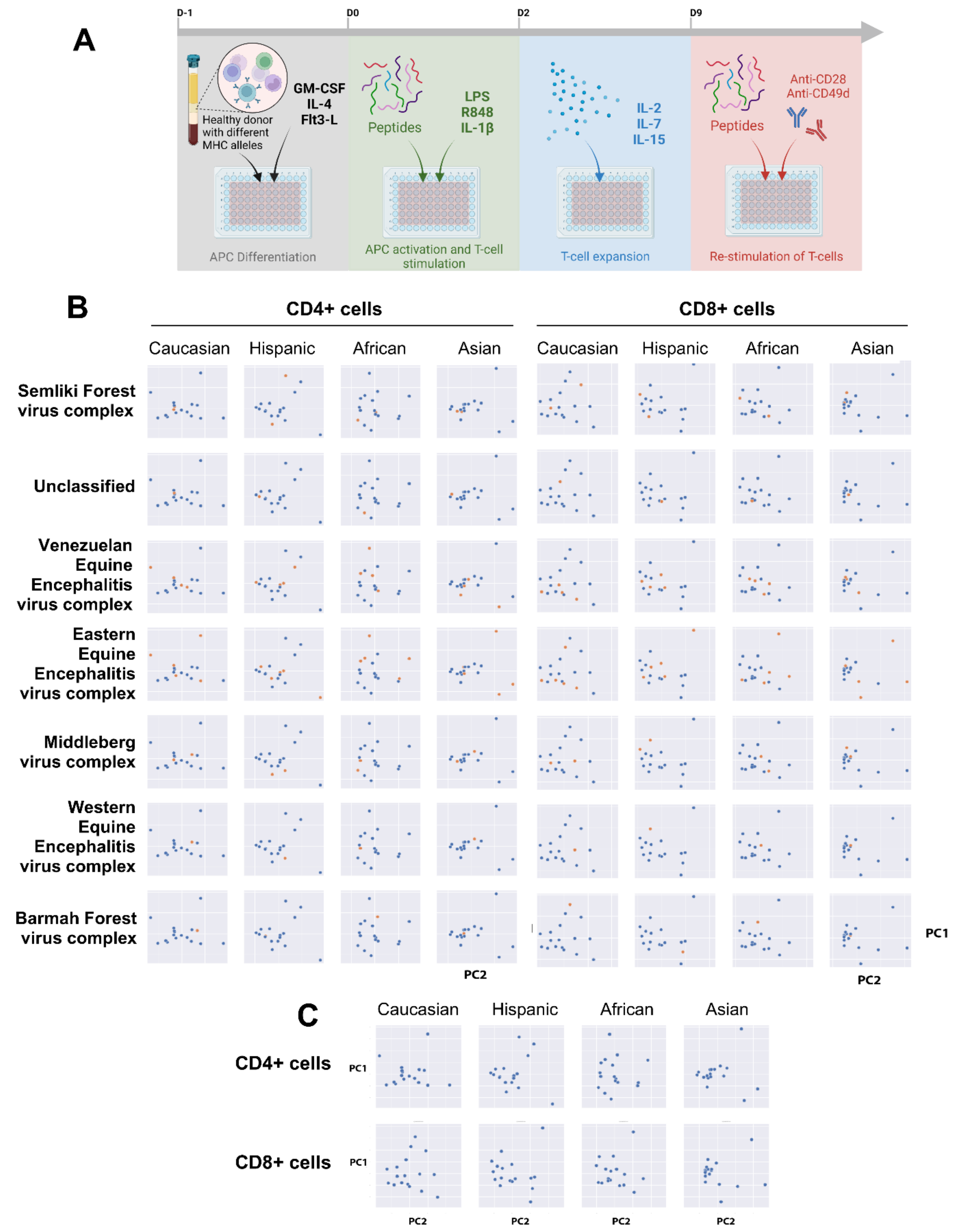

Figure S9. Immunogenic profiles of PBMCs from healthy donors after in vitro expansion with pan-alphaviruses peptides. (A) Schematic design of peptide-specific in vitro T-cell differentiation and clonal expansion protocol described in (29). The following plots represent a Principal Components Analysis (PCA) performed on each of the 17 epitopes. In these plots, the first two principal components (PC1 and PC2) constitute the y and x axes respectively and each point represents a single epitope. Input to PCA analysis were cytokine and T-cell activation markers measurements using donor PBMCs. In total there were six signatures measured: CD107a, CD137, CD154, IFN-gamma, TNF, and IL-2 for alongside CD4 and CD8, compressing 6 dimensions into two principal components. The different plots show these data filtered by (B) CD4 and CD8, as well as (C) ethnicity or virus complex prior to PCA.

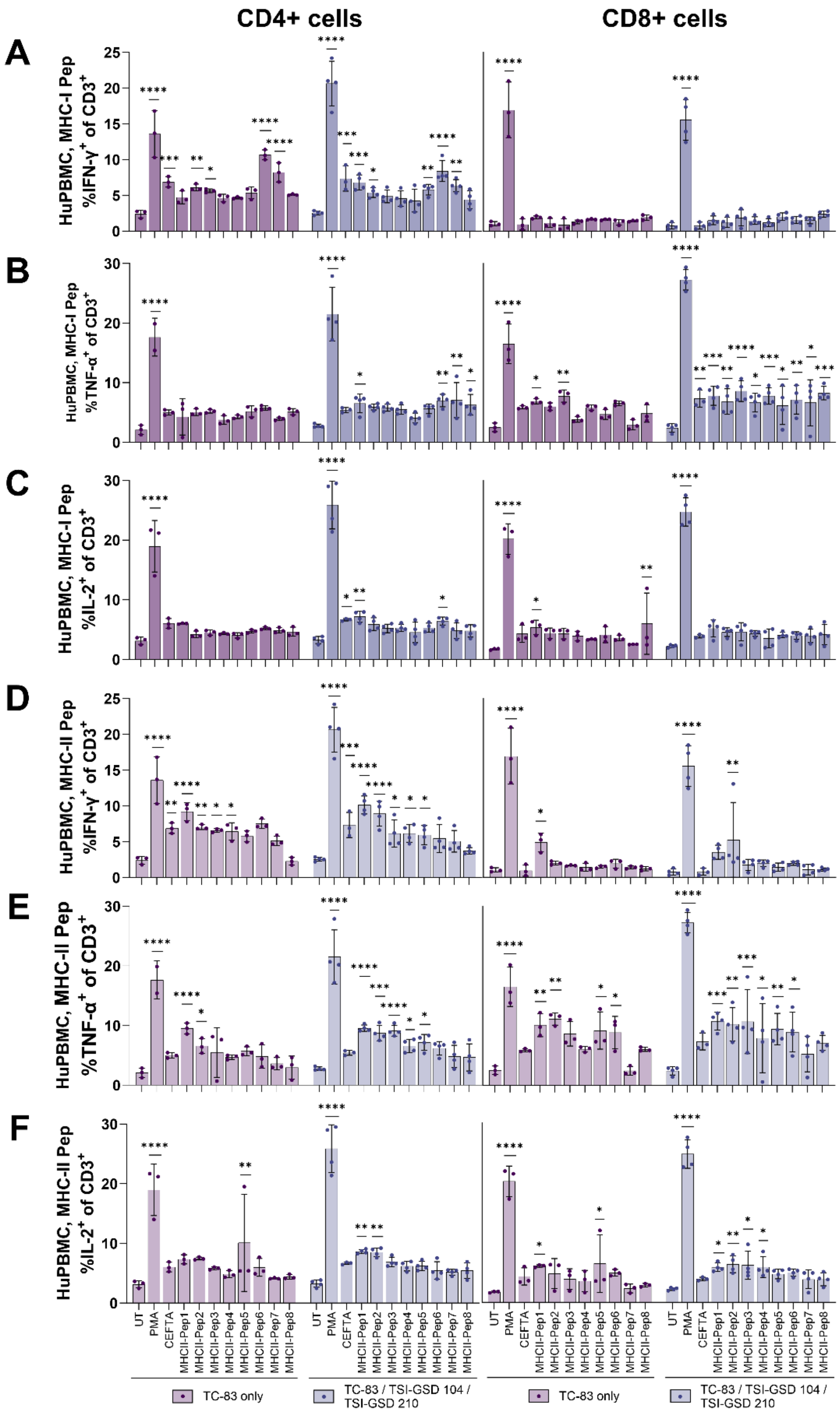

Figure S10. Cytokine induction on human PBMCs followed by *in vitro* stimulation with alphaviruses peptides. PBMCs from donors previously exposed to alphaviruses vaccines were stimulated with the vehicle control (UT, untreated), positive control (PMA), CEFT (which contains known viral epitopes), and test alphaviruses peptides. (A) MHC-I peptides, IFN-gamma+ cells. (B) MHC-I peptides, TNF-alpha+ cells. (C) MHC-I peptides, IL-2+ cells. (D) MHC-II peptides, IFN-gamma+ cells. (E) MHC-II peptides, TNF-alpha+ cells. (F) MHC-II peptides, IL-2+ cells. Left graphs indicate CD4+ cells, right graphs indicate CD8+ cells. Percentage of T-cells was measured by flow cytometry. Data were represented as mean ± standard deviation (SD). p-values obtained by 2-way ANOVA with Dunnett's multiple comparisons test. (*) p<0.5; (**) p<0.01; (***) p>0.001; (****) p<0.0001.

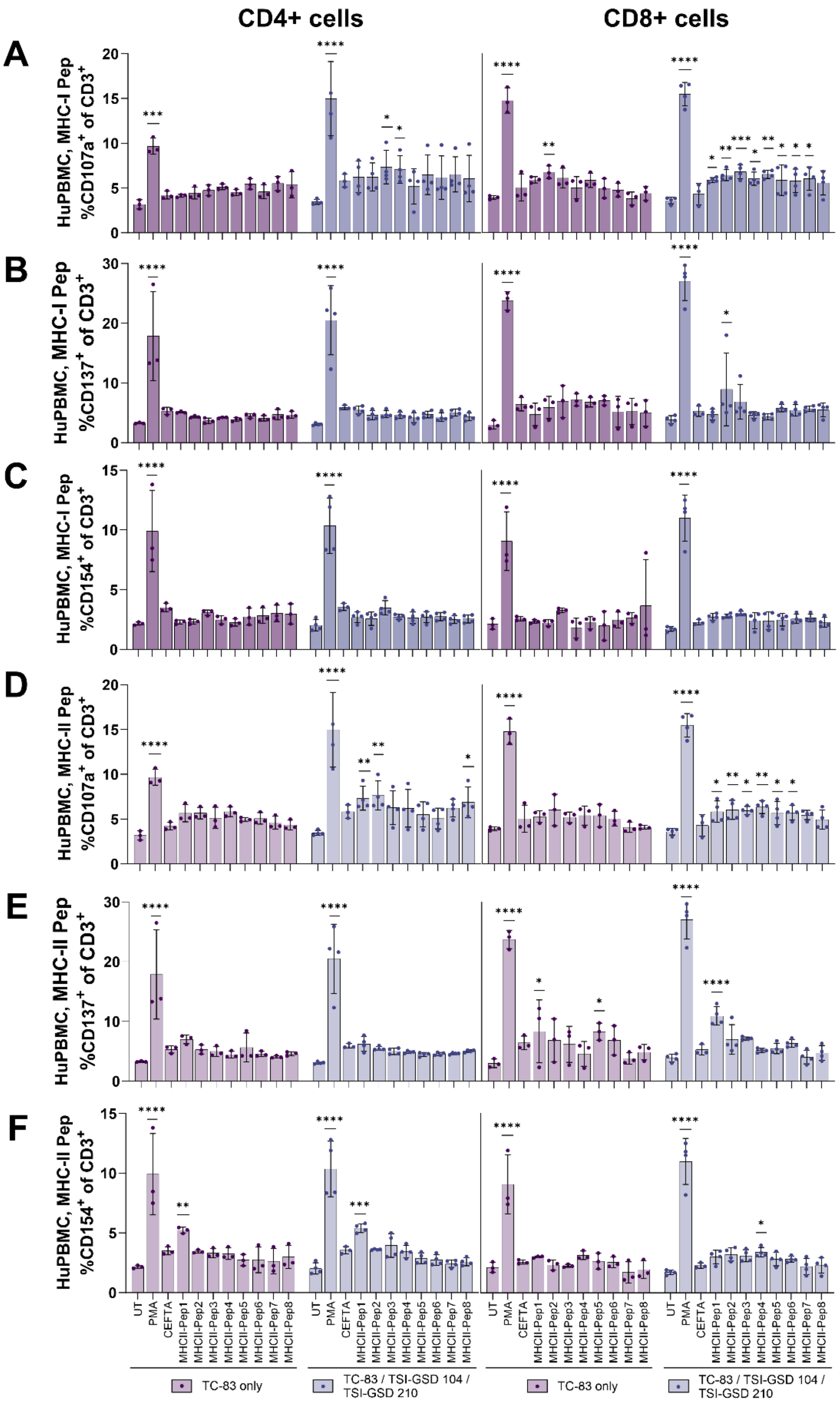

Figure S11. Surface markers induction on human PBMCs followed by *in vitro* stimulation with alphaviruses peptides. PBMCs from donors previously exposed to alphaviruses vaccines were stimulated with the vehicle control (UT, untreated), positive control (PMA), CEFT (which contains known viral epitopes), and test alphaviruses peptides. (A) MHC-I peptides, CD107a+ cells. (B) MHC-I peptides, CD137+ cells. (C) MHC-I peptides, CD154+ cells. (D) MHC-II peptides, CD107a+ cells. (E) MHC-II peptides, CD137+ cells. (F) MHC-II peptides, CD154+ cells. Left graphs indicate CD4+ cells, right graphs indicate CD8+ cells. Percentage of T-cells was measured by flow cytometry. Data were represented as mean ± standard deviation (SD). p-values obtained by 2-way ANOVA with Dunnett's multiple comparisons test. (*) p<0.5; (**) p<0.01; (***) p>0.001; (****) p<0.0001.

Table S1. **List of accession numbers for viral proteomes used for the epitope selection.** Name includes the viral representative for each accession number.

Accession	Name
AB032553_1	Getah Virus
AF075251_1	Everglades Virus
AF075253_1	Mucambo Virus
AF075254_1	Tonate Virus
AF075255_1	Venezuelan Equine Encephalitis Virus
AF075256_1	Pixuna Virus
AF075257_1	Mosso Pas Pedras Virus
AF075258_1	Rio Negro Virus
AF075259_1	Cabassou Virus
AF079456_1	Onyong-nyong Virus
AF079457_1	Onyong-nyong Virus
AF103728_1	Sindbis Virus
AF126284_1	Aura Virus
AF214040_1	Western Equine Encephalitis Virus
AF237947_1	Mayaro Virus
AF252265_1	Trocara Virus
AF369024_2	Chikungunya Virus
AF375051_1	Venezuelan Equine Encephalitis Virus
AF429428_1	Sindbis Virus
AJ316246_1	Salmon Pancreas Disease Virus
AY702913_1	Getah Virus
DQ241303_1	Madariaga Virus
EF011023_1	Getah Virus
EF151503_1	Madariaga Virus
EF536323_1	Middelburg Virus
FJ827631_1	Highlands J Virus
GQ281603_1	Fort Morgan Virus
GQ287646	Western Equine Encephalitis Virus
GQ433354_1	Ross River Virus
HM147984_1	Sindbis Virus
HM147985_1	Bebaru Virus
HM147986_1	Fort Morgan Virus
HM147989_1	Ndumu Virus
HM147990_1	Southern Elephant Seal Virus
HM147991_1	Trocara Virus
HM147992_1	Una Virus
HM147993_1	Whataroa Virus
J02363_1	Sindbis Virus
JF972635_1	Semliki Forest Virus
JX678730_1	Eilat Virus
KJ469640_1	Madariaga Virus
KM115530_1	Middelburg Virus

KP003813_2	Chikungunya Virus
L00930_1	Venezuelan Equine Encephalitis Virus
L01442_2	Venezuelan Equine Encephalitis Virus
M20162_1	Ross River Virus
M20303_1	Onyong-nyong Virus
M69205_1	Sindbis Virus
MK353339_2	Caaingua Virus
U34999_1	Venezuelan Equine Encephalitis Virus
U73745_1	Barmah Forest Virus
X04129_1	Semliki Forest Virus
X63135_1	Eastern Equine Encephalitis Virus

Table S2. Detailed list of sera banks. Mouse, non-human primate, and human sera banks used for epitope microarray reactivity analysis.

Species	Virus	Sample ID	Additional info
Mouse	Naïve	ABSL2#56 m1	PRNT negative TC-83/MAYV/ZIKV
		ABSL2#56 m2	PRNT negative TC-83/MAYV/ZIKV
		ABSL2#56 m3	PRNT negative TC-83/MAYV/ZIKV
		ABSL2#56 m4	PRNT negative TC-83/MAYV/ZIKV
		ABSL2#56 m5	PRNT negative TC-83/MAYV/ZIKV
	ZIKV	ABSL2#56 m16	$PRNT_{50}$ >1:640 / $PRNT_{80}$ >1:640.
		ABSL2#56 m17	$PRNT_{50}$ >1:640 / $PRNT_{80}$ >1:640.
		ABSL2#56 m18	$PRNT_{50}$ >1:640/$PRNT_{80}$ >1:640.
		ABSL2#56 m19	$PRNT_{50}$ >1:640/$PRNT_{80}$ >1:640.
		ABSL2#56 m20	$PRNT_{50}$ >1:640/$PRNT_{80}$ >1:640.
		ABSL2#56 m21	$PRNT_{50}$ >1:640/$PRNT_{80}$ >1:640.
	VEEV	ABSL2#51a m29	$PRNT_{50}$ >1:640 / $PRNT_{80}$ >1:640
		ABSL2#51a m30	$PRNT_{50}$ >1:640 / $PRNT_{80}$ >1:640
		ABSL2#51b m17	$PRNT_{50}$ >1:640 / $PRNT_{80}$ >1:640
		ABSL2#51b m19	$PRNT_{50}$ >1:640 / $PRNT_{80}$ >1:640
	MAYV	Study1 m17	36dpi. $PRNT_{50}$ >1:640/$PRNT_{80}$ >1:640.
		Study1 m22	36dpi. $PRNT_{50}$ 1:40/$PRNT_{80}$ <1:20.
	CHIKV	m1	$PRNT_{50}$ >1:640 / $PRNT_{80}$ >1:640
		m2	$PRNT_{50}$ >1:640 / $PRNT_{80}$ >1:160
NHP	Naïve	Cynomolgus naïve	Pre-immune serum.
		Saimiri naïve	Pre-immune serum.
	ZIKV	ABLS2#1912100 NHP6550	28dpi. $PRNT_{50}$ 1:160 / $PRNT_{80}$ 1:80.
		ABLS2#1912100 NHP4806	28dpi. $PRNT_{50}$ 1:80 / $PRNT_{80}$ <1:20.
	CHIKV	MV-CHIKV-204 pool high	$PRNT_{50}$ >1:640 / $PRNT_{80}$ 1:160
Human	Naïve	Control serum (reference sample)	Millipore Sigma, REF #NIST® SRM® 909c
	VEEV*	702193	$PRNT_{80}$ 1:80
		702196	$PRNT_{80}$ 1:160
		702218	$PRNT_{80}$ 1:320
		702228	$PRNT_{80}$ 1:160
		702229	$PRNT_{80}$ 1:80
		702241	$PRNT_{80}$ 1:640
	MADV*	702216	$PRNT_{80}$ 1:640
		702226	$PRNT_{80}$ 1:160
		702231	$PRNT_{80}$ 1:20
		702232	$PRNT_{80}$ 1:640
	CHIKV[#]	A02 307	$PRNT_{80}$ >1:20
		A02 537	$PRNT_{80}$ >1:20
		A02 560	$PRNT_{80}$ >1:20
		FB 307	$PRNT_{80}$ >1:20
		A01 307	$PRNT_{80}$ >1:20
		A01 54	$PRNT_{80}$ >1:20
		A01 712	$PRNT_{80}$ >1:20

		A01 560	$PRNT_{80}$ >1:20
		A01 447	$PRNT_{80}$ >1:20
		A02 48	$PRNT_{80}$ >1:20
		A02 54	$PRNT_{80}$ >1:20
		A02 712	$PRNT_{80}$ >1:20
		A02 799	$PRNT_{80}$ >1:20
		A02 961	$PRNT_{80}$ >1:20
		A02 1127	$PRNT_{80}$ >1:20
		A02 554	$PRNT_{80}$ >1:20

* $PRNT_{80}$ were performed at Instituto Conmemorativo Gorgas de Estudios de la Salud (ICGES), October 2018.

Initial $PRNT_{80}$ were performed at Departamento de Doenças Infecciosas, Faculdade de Medicina de São José do Rio Preto, São José do Rio Preto, SP, Brazil, and first reported in Zini etal "Cryptic circulation of chikungunya virus in São Jose do Rio Preto, Brazil, 2015-2019". *PLoS NTD* 18(3):e0012013; 2024 *https://doi.org/10.1371/journal.pntd.0012013*. The $PRNT_{80}$ values reported here, were performed at the Vasilakis laboratory at the University of Texas Medical Branch, TX, USA

Table S3. Final selected MHC-I and MHC-II epitopes. Each ID is classified by T-cell type used for selection, presence in B-cell cluster, species, root, and epitope protein position.

ID	Sequence	T-cell type	B-cell cluster?	Classification/Species	Root	Protein position
MHC-I Pep1	APLQHTAPF	MHC-I	No	top_ranked human_primate_mouse	Chikungunya virus; Onyong-nyong virus; Igbo Ora virus	E1
MHC-I Pep2	APRRRVGGF	MHC-I	No	top_ranked human_primate_mouse	Madariaga virus; Eastern equine encephalitis virus	nsp3
MHC-I Pep3	FPSISTTAW	MHC-I	No	top_ranked human_primate_mouse	Barmah Forest virus	E1
MHC-I Pep4	HPQHHAQTF	MHC-I	No	top_ranked human_primate_mouse	Venezuelan equine encephalitis virus; Tonate virus; Mucambo virus; Cabassou virus; Everglades virus	E1
MHC-I Pep5	HPQLHAQTF	MHC-I	No	top_ranked human_primate_mouse	Venezuelan equine encephalitis virus; Tonate virus; Mucambo virus; Cabassou virus; Everglades virus	E1
MHC-I Pep6	KPDYRCQTY	MHC-I	No	top_ranked human_primate_mouse	Una virus; Semliki Forest virus; Trocara virus; Sagiyama virus; Getah virus; Yada yada virus; Caaingua virus	E1
MHC-I Pep7	PCCYEKGPE	MHC-I	Yes	no_mouse_no_primate_ no_human	Chikungunya virus; Mayaro virus; Ross River virus; Onyong-nyong virus; Igbo Ora virus	E3
MHC-I Pep8	PCCYEKQPE	MHC-I	Yes	no_mouse_no_primate_ no_human	Chikungunya virus; Ross River virus; Onyong-nyong virus; Sagiyama virus; Getah virus	E3
MHC-I Pep9	PDDQDTGSE	MHC-I	Yes	no_mouse_no_primate_ no_human	Sleeping disease virus; Salmonid alphavirus subtype 3	nsp4
MHC-II Pep1	APCSLVSYHGYYILA	MHC-II	Yes	human_top_mouse_bottom	Rio Negro virus; Venezuelan equine encephalitis virus; Pixuna virus; Madariaga virus; Eastern equine encephalitis virus	E2
MHC-II Pep2	CYMFATARRKCLTPY	MHC-II	No	top_ranked_human_mouse	Bebaru virus; Una virus; Chikungunya virus; Middelburg virus; Mayaro virus; Semliki Forest virus; Western equine encephalitis virus	E2
MHC-II Pep3	HAGYIRIQTSAMFGL	MHC-II	No	top_ranked_human_mouse	Venezuelan equine encephalitis virus; Tonate virus; Mucambo virus; Cabassou virus; Madariaga virus; Eastern equine encephalitis virus	E2
MHC-II Pep4	IIFVNMRTPYKHHHY	MHC-II	No	top_ranked_human_mouse	Venezuelan equine encephalitis virus; Madariaga virus; Eastern equine encephalitis virus	nsp2
MHC-II Pep5	KALITQRMLKGLGHY	MHC-II	No	top_ranked_human_mouse	Rio Negro virus; Venezuelan equine encephalitis virus; Pixuna virus; Madariaga virus; Eastern equine encephalitis virus	nsp4
MHC-II Pep6	KLFLAKSATRSIVER	MHC-II	No	top_ranked_human_mouse	Bebaru virus	nsp2
MHC-II Pep7	LARRFSSFRAVTVRC	MHC-II	No	top_ranked_human_mouse	Mayaro virus; Ross River virus; Sagiyama virus; Getah virus	E2
MHC-II Pep8	LASCYMFATARRKCL	MHC-II	No	top_ranked_human_mouse	Una virus; Middelburg virus; Mayaro virus; Semliki Forest virus; Ross River virus; Sagiyama virus; Getah virus	E2

Table S4. Analysis of peptide models bound to MHC. Generated by AlphaFold2-Multimer (AF2M) or ColabFold.

MHC	MHC alelle	peptide	Comparative peptide backbone RMSD (Å) / PDB reference	Confidence score
Class I	A*02	pep1	1.8 / 5eu4 (AF2M)<br>1.8 / 5eu4 (ColabFold)	0.92 (AF2M)<br>0.92 (ColabFold)
		pep2	4.1 / 2gtw (AF2M)<br>0.8 / 5eu4 (ColabFold)	0.68 (AF2M)<br>0.79 (ColabFold)
		pep3	2.1 / 2gtw (AF2M)<br>1.7 / 2gtw (ColabFold)	0.60 (AF2M)<br>0.6 (ColabFold)
		pep4	0.6 / 3ft4 (AF2M)<br>0.6 / 5eu5 (ColabFold)	0.91 (AF2M)<br>0.87 (ColabFold)
		pep5	0.6 / 3ft4 (AF2M)<br>0.6 / 5hhn (ColabFold)	0.91 (AF2M)<br>0.88 (ColabFold)
		pep6	0.6 / 5hhn (AF2M)<br>0.6 / 6ptb (ColabFold)	0.74 (AF2M)<br>0.66 (ColabFold)
		pep7	13.7 / 2gtw (AF2M)<br>1.9 / 2x4s (ColabFold)	0.85 (AF2M)<br>0.68 (ColabFold)
		pep8	2.6 / 2v2x (AF2M)<br>2.23 / 2v2x (ColabFold)	0.75 (AF2M)<br>0.73 (ColabFold)
		pep9	1.2 / 7lg2 (AF2M)<br>1.9 / 1qr1 (ColabFold)	0.74 (AF2M)<br>0.68 (ColabFold)
Class II	DRA1: DRB1*07:01:01:01	pep1	1.2 / 3l6f (AF2M)<br>2.4 / 3l6f (ColabFold)	0.91 (AF2M)<br>0.87 (ColabFold)
		pep2	2.1 / 1klg (AF2M)<br>1.7 / 4z7u (ColabFold)	0.89 (AF2M)<br>0.87 (ColabFold)
		pep3	1.8 / 6blx (AF2M)<br>2.3 / 6blx (ColabFold)	0.87 (AF2M)<br>0.85 (ColabFold)
		pep4	4.2 / 4z7u (AF2M)<br>3.8 / 1sje (ColabFold)	0.86 (AF2M)<br>0.85 (ColabFold)
		pep5	1.3 / 2ian (AF2M)<br>1.1 / 2ian (ColabFold)	0.87 (AF2M)<br>0.88 (ColabFold)
		pep6	2.9 / 3cup (AF2M)<br>1.3 / 2ian (ColabFold)	0.87 (AF2M)<br>0.92 (ColabFold)
		pep7	1.5 / 6blx (AF2M)<br>1.5 / 6blx (ColabFold)	0.92 (AF2M)<br>0.90 (ColabFold)
		pep8	6.1 / 6blx (AF2M)<br>10.7 / 6blx (ColabFold)	0.85 (AF2M)<br>0.87 (ColabFold)

Peptides were modeled bound to the corresponding MHC alleles (A*02 or DRA1: DRB1*07:01:01:01). The feasibility of modeled binding was assessed by comparing peptide backbone structures to a reference set of 3D structures of peptides bound to MHC-I or MHC-II molecules. The table includes the lowest observed deviation, assessed by the root-mean-square deviation (RMSD), along with the corresponding PDB reference structure. Additionally, confidence scores provided by AF2M or ColabFold are listed. This score corresponds to the combination of ipTM and pTM scores (0.8*ipTM + 0.2*pTM), as described in *(25).* For MHC-II molecules, the provided AF2M or ColabFold scores incorporate the interface between the MHC chains in the calculation by default.

Table S5. Identification and prediction of the binding cores of the MHC-II bound peptides.

Peptide	Peptide core in 3D model	NetMHCIIpan core prediction / reliability score
MHCII_pep1	APCS**LVSYHGYYI**LA	APCS**LVSYHGYYI**LA / 0.972
MHCII_pep2	CYM**FATARRKCL**TPY	C**YMFATARRK**CLTPY / 0.533
MHCII_pep3	HAGY**IRIQTSAMF**GL	HAG**YIRIQTSAM**FGL / 0.767
MHCII_pep4	NA	II**FVNMRTPYK**HHHY / 0.593
MHCII_pep5	KAL**ITQRMLKGL**GHY	KAL**ITQRMLKGL**GHY / 0.407
MHCII_pep6	KLF**LAKSATRSI**VER	KLF**LAKSATRSI**VER / 0.98
MHCII_pep7	LARR**FSSFRAVTV**RC	LARR**FSSFRAVTV**RC / 1.0
MHCII_pep8	NA	LASC**YMFATARRK**CL / 0.927

The peptide binding cores are highlighted in bold. The peptide core in the 3D models generated by AF2M or ColabFold was assessed by visual inspection of MHC-II anchor sites. NA(‘not applicable’) indicates peptides that could not be corrected modeled. Alongside the core sequence predicted by NetMHCIIpan, the reliability score of the binding core, expressed as the fraction of networks in the ensemble *(28)*, is also presented.

Table S6. PMBC healthy donors' descriptives, including ethnicity, age, gender, and HLA-typing. Descriptives were provided by vendor (ImmunoSpot, USA).

	Donor #	1	2	3	4	5	6	7	8	9	10	11	12
Demographics	Sample ID #	HHU20220602	HHU20210202	HHU20211221	HHU20191212	HHU20200305	HHU20200428	HHU20200507	HHU20200213	HHU20220825	HHU20210831	HHU20211007	HHU20230727
	Collection Date	6/1/2022	2/1/2021	12/20/2021	6/29/2020	3/4/2020	4/28/2020	5/6/2020	2/12/2020	3/6/2023	8/30/2021	10/6/2021	7/25/2023
	Ethnicity	Caucasian	Caucasian	Caucasian	Hispanic	Hispanic	Hispanic	African / American	African / American	African / American	Asian	Asian	Asian
	Age	38	31	36	39	54	51	30	58	48	21	22	34
	Gender	Female	Female	Female	Male	Male	Male	Male	Male	Male	Male	Male	Female
	ABO/Rh	B/Pos	0/Neg	0/Pos	A/Pos	0/Neg	0/Pos	A/Pos	0/Pos	0/Pos	A/Pos	A/Pos	B/Pos
HLA Class I	HLA-A	A*02:01 / A*24:02	A*02:01 / A*03:01	A*02:01 / A*03:01	A*01:01 / A*68:01	A*02:01 / A*26:01	A*02:01 / A*24:03	A*23:01 / A*33:01	A*30:01 / A*30:01	A*30:02 / A*33:03	A*31:01 / ~	A*01:01 / A*32:01	A*03:01 / A*24:30
	HLA-B	B*07:02 / B*15:01	B*07:02 / B*44:02	B*07:02 / B*57:01	B*08:01 / B*15:40	B*14:01 / B*35:01	B*35:01 / B*35:12	B*07:06 / B*42:01	B*42:01 / B*42:01	B*08:01 / B*58:01	B*15:01 / B*44:03	B*35:01 / B*40:06	B*38:02 / B*57:01
	HLA-C	C*03:03 / C*07:02	C*05:01 / C*07:02	C*06:02 / C*07:02	C*03:03 / C*07:01	C*04:01 / C*08:02	C*04:01 / C*04:01	C*07:02 / C*17:01	C*17:01 / C*17:01	C*07:01 / C*07:18	C*03:04 / C*14:03	C*04:01 / C*15:02	C*06:02 / C*07:02
HLA Class II	HLA-DRB1	DRB1*04:01 / DRB1*04:04	DRB1*04:01 / DRB1*15:01	DRB1*07:01 / DRB1*15:01	DRB1*03:01 / DRB1*08:02	DRB1*07:01 / DRB1*16:02	DRB1*08:02 / DRB1*13:01	DRB1*01:01 / DRB1*03:02	DRB1*03:02 / DRB1*11:02	DRB1*03:01 / DRB1*15:03	DRB1*11:01 / DRB1*13:02	DRB1*15:02 / DRB1*16:02	DRB1*07:01 / DRB1*12:02
	HLA-DQB1	DQB1*03:02 / ~	DQB1*03:01 / DQB1*06:02	DQB1*03:03 / DQB1*06:02	DQB1*02:01 / DQB1*04:02	DQB1*02:02 / DQB1*03:01	DQB1*04:02 / DQB1*06:03	DQB1*04:02 / DQB1*05:01	DQB1*03:19 / DQB1*04:02	DQB1*02:01 / DQB1*06:02	DQB1*02:01 / DQB1*06:04	DQB1*05:02 / DQB1*06:01	DQB1*03:03 / DQB1*05:02
	HLA-DPB1	DPB1*04:01 / DPB1*05:01	DPB1*04:01 / DPB1*11:01	DPB1*04:01 / ~	DPB1*04:01 / DPB1*105:01	DPB1*02:01G / DPB1*04:02G	DPB1*04:02 / DPB1*19:01	DPB1*01:01 / DPB1*85:01	DPB1*01:01G / DPB1*85:01G	DPB1*02:01 / DPB1*02:01	DPB1*02:01 / DPB1*04:01	DPB1*02:01 / DPB1*04:01	DPB1*02:01 / DPB1*21:01
	HLA-DQA1	DQA1*03:01 / ~	DQA1*01:02 / DQA1*03:01	DQA1*01:02 / DQA1*02:01	DQA1*04:01 / DQA1*05:01	not tested	DQA1*01:03 / DQA1*04:01	DQA1*01:01 / DQA1*04:01	not tested	DQA1*01:02 / DQA1*05:01	DQA1*01:02 / DQA1*05:01	DQA1*01:02 / DQA1*01:03	DQA1*02:02 / DQA1*02:01
	HLA-DRB3/4/5	DRB4*01:01 / ~	DRB4*01:01 / DRB5*01:01	DRB4*01:01 / DRB5*01:01	DRB3*01:01 / ~	DRB4*01:01 / DRB5*02:02	DRB3*02:02 / ~	DRB3*01:01 / ~	DRB3*01:01 / DRB3*02:02	DRB3*02:02 / DRB5*01:01	DRB3*02:02 / DRB3*03:01	DRB5*01:02 / DRB5*02:02	DRB3*03:01 / DRB4*01:03
	HLA-DPA1	DPA1*01:03 / DPA1*02:02	DPA1*01:03 / DPA1*02:01	DPA1*01:03 / ~	DPA1*01:03 / DPA1*01:03	DPA1*01:03 / DPA1*01:03	DPA1*01:03 / DPA1*02:07	DPA1*02:01 / DPA1*02:12	DPA1*02:02 / DPA1*02:12	DPA1*01:03 / DPA1*01:03	DPA1*01:03 / ~	DPA1*01:03 / ~	DPA1*01:03 / DPA1*01:03
CD16-V212F		not tested	not tested	not tested	CD16-Phe/Phe	CD16-Val/Phe	CD16-Val/Phe	CD16-Phe/Phe	CD16-Val/Val	not tested	not tested	not tested	not tested

Table S7. PBMC donors pre-exposed to alphaviruses proteins. List of demographics and vaccination status.

	Donor #	1	2	3	4	5	6	7	8
Demographics	Sample ID #	mcm_0001	mcm_0002	mcm_0003	mcm_0004	mcm_0005	mcm_0006	mcm_0007	mcm_0008
	Collection Date	1/1/2024	1/1/2024	1/1/2024	1/1/2024	1/1/2024	1/1/2024	1/1/2024	1/1/2024
	Ethnicity	Caucasian	Caucasian	Caucasian	African / American	Caucasian	Caucasian	Caucasian	Caucasian
	Age	43	39	35	40	38	38	66	41
	Gender	Female	Male	Male	Male	Female	Male	Male	Male
Vaccine status	TC-83: live attenuated VEEV vaccine	✓	✓	✓	✓	✓	✓	✓	✓
	TSI-GSD 104/inactivated PE-6 strain: EEEV vaccine		✓	✓	✓			✓	✓
	TSI-GSD 210/inactivated CM-4884 strain: WEEV vaccine		✓	✓	✓			✓	✓
TC-83 neutralization titer		1:40	1:160	1:320	1:320	1:20	>1:640	>1:640	>1:640

Table S8. Flow cytometry markers used on mouse and human cocktails.

Species	Type	Marker	Clone	Cat #	Brand
Mouse	Surface	PerCP/Cyanine 5.5 anti-mouse CD3ε	145-2C11	100326	Biolegend
		Brilliant Violet 510™ anti-mouse CD4	RM4-4	116025	Biolegend
		Alexa Fluor® 700 anti-mouse CD8a	53-6.7	100730	Biolegend
		APC anti-mouse CD137	17B5	106110	Biolegend
		Brilliant Violet 421™ anti-mouse CD25	PC61	102043	Biolegend
		PE anti-mouse CD134 (OX-40)	OX-86	119409	Biolegend
		PE/Cyanine7 anti-mouse CD69	H1.2F3	104512	Biolegend
	Intracellular cytokine	Brilliant Violet 711™ anti-mouse IFN-γ	XMG1.2	505836	Biolegend
		PE/Dazzle™ 594 anti-mouse TNF-α	MP6-XT22	506346	Biolegend
		Brilliant Violet 605™ anti-mouse IL-2	JES6-5H4	503829	Biolegend
Human	Surface	FITC anti-human CD3	SK7	344803	Biolegend
		Brilliant Violet 785™ anti-human CD4	RPA-T4	300554	Biolegend
		APC anti-human CD8	RPA-T8	301049	Biolegend
		PerCP/Cyanine5.5 anti-human CD137 (4-1BB)	4B4-1	309814	Biolegend
		Brilliant Violet 421™ anti-human DC107a (LAMP-1)	H4A3	328626	Biolegend
		Brilliant Violet 711™ anti-human CD154	24-31	310838	Biolegend
	Intracellular cytokine	PE anti-human IFN-γ	B27	506507	Biolegend
		PE/Cyanine 7 anti-human TNF-α	MAb11	502930	Biolegend
		Brilliant Violet 605™ anti-human IL-2	MQ1-17H12	500332	Biolegend
Live/dead		Zombie NIR™ Fixable Viability Kit	-	423106	Biolegend
